# Requirements for Receptor Engagement during Infection by Adenovirus Complexed with Blood Coagulation Factor X

**DOI:** 10.1371/journal.ppat.1001142

**Published:** 2010-10-07

**Authors:** Angela C. Bradshaw, Alan L. Parker, Margaret R. Duffy, Lynda Coughlan, Nico van Rooijen, Veli-Matti Kähäri, Stuart A. Nicklin, Andrew H. Baker

**Affiliations:** 1 Institute of Cardiovascular and Medical Sciences, British Heart Foundation Glasgow Cardiovascular Research Centre, College of Medical, Veterinary and Life Sciences, University of Glasgow, Glasgow, United Kingdom; 2 Department of Molecular Cell Biology, Vrije Universiteit Medical Center (VUMC), Amsterdam, The Netherlands; 3 Department of Dermatology, University of Turku and Turku University Central Hospital, Turku, Finland; University of Michigan, United States of America

## Abstract

Human adenoviruses from multiple species bind to coagulation factor X (FX), yet the importance of this interaction in adenovirus dissemination is unknown. Upon contact with blood, vectors based on adenovirus serotype 5 (Ad5) binds to FX via the hexon protein with nanomolar affinity, leading to selective uptake of the complex into the liver and spleen. The Ad5:FX complex putatively targets heparan sulfate proteoglycans (HSPGs). The aim of this study was to elucidate the specific requirements for Ad5:FX-mediated cellular uptake in this high-affinity pathway, specifically the HSPG receptor requirements as well as the role of penton base-mediated integrin engagement in subsequent internalisation. Removal of HS sidechains by enzymatic digestion or competition with highly-sulfated heparins/heparan sulfates significantly decreased FX-mediated Ad5 cell binding *in vitro* and *ex vivo*. Removal of *N*-linked and, in particular, *O*-linked sulfate groups significantly attenuated the inhibitory capabilities of heparin, while the chemical inhibition of endogenous HSPG sulfation dose-dependently reduced FX-mediated Ad5 cellular uptake. Unlike native heparin, modified heparins lacking *O*- or *N*-linked sulfate groups were unable to inhibit Ad5 accumulation in the liver 1h after intravascular administration of adenovirus. Similar results were observed *in vitro* using Ad5 vectors possessing mutations ablating CAR- and/or α_v_ integrin binding, demonstrating that attachment of the Ad5:FX complex to the cell surface involves HSPG sulfation. Interestingly, Ad5 vectors ablated for α_v_ integrin binding showed markedly delayed cell entry, highlighting the need for an efficient post-attachment internalisation signal for optimal Ad5 uptake and transport following surface binding mediated through FX. This study therefore integrates the established model of α_v_ integrin-dependent adenoviral infection with the high-affinity FX-mediated pathway. This has important implications for mechanisms that define organ targeting following contact of human adenoviruses with blood.

## Author Summary

Adenoviruses can infect many cell types and cause a range of illnesses in humans, including respiratory, ocular and gastrointestinal disorders. These illnesses are rarely fatal; however, in immunocompromised individuals, especially young children, disseminated adenovirus infections can cause serious and life-threatening complications. Studies have shown that several adenoviruses including vectors based on adenovirus serotype 5 (Ad5) bind to coagulation factor X (FX) in the bloodstream. Ad5 uses the high-affinity interaction with FX to putatively bind to heparan sulfate proteoglycans (HSPGs). However, very little is known about this infection pathway. Here we demonstrate that interaction of Ad5:FX with HSPGs is solely via the HS sidechains of these ubiquitously-expressed molecules. We further show that this interaction is dependent on HS sulfation, in particular *O*-sulfation. Although attachment of Ad5:FX to HSPGs is independent of the coxsackievirus and adenovirus receptor (CAR) or α_v_ integrins, efficient and rapid intracellular transport of Ad5 retains a dependence on engagement of α_v_ integrins via the penton base protein. This is the first study to characterise the receptor requirements for cell uptake via the recently-identified, FX-mediated infection pathway, which may be of significance for the development of therapies against disseminated adenoviral disease.

## Introduction

Adenoviruses are non-enveloped, icosahedral double-stranded DNA viruses of 70–90nm diameter. 54 different human serotypes have been identified to date and are classified into species based on their ability to agglutinate human, monkey or rat erythrocytes [1]. Adenoviruses cause a range of illnesses depending on the route of initial infection, largely dictated by inherent adenoviral tropism. These illnesses are usually self-limiting but can become potentially life-threatening in certain circumstances. For example, species C adenoviruses 1, 2 and 5 initially cause respiratory tract infections after inhaled droplet transmission [2] but are associated with fulminant hepatitis in bone marrow transplant patients [3], [4]. Invasive adenovirus infection following liver transplant is relatively common, occurring in approximately 10% of paediatric and 6% of adult liver transplantion recipients (reviewed in [5]) and may be due to latent donor-associated infection of the transplanted organ. Adenovirus has also been detected in peripheral blood from immunocompromised patients [6], [7], [8], [9] a significant proportion of whom then go on to develop potentially fatal disseminated adenoviral disease. Taken together, these studies underline the clinical significance of these common human pathogens. The primary and secondary receptor systems used by adenoviruses for cellular uptake following contact with different environments *in vivo* are thus of particular interest and importance.

The species C adenovirus Ad5 can efficiently infect a wide variety of cell types. *In vitro* studies have demonstrated that cell tethering is mediated by a primary interaction of the Ad5 fiber knob domain with the coxsackievirus and adenovirus receptor (CAR; [10], reviewed in [11]), while the subsequent internalisation of Ad5 particles is dependent on binding of α_v_β_3_/α_v_β_5_ integrins to an RGD motif in the adenovirus penton base [12], [13]. Several *in vivo* studies, however, have shown that direct interaction with CAR is not required for uptake of Ad5 into the liver [14], [15], [16], [17], which is the primary target organ after contact of adenovirus with the bloodstream in rodent models and in non-human primates (reviewed in [18]). Moreover, CAR is now thought to be localised primarily to tight junctions in intact epithelium, rendering it inaccessible to viral particles (reviewed in [19]). Instead, recent studies have demonstrated that uptake of Ad5 into the liver is mediated by a high affinity interaction with blood coagulation factor X (FX), which putatively ‘bridges’ the hexon protein in the adenovirus capsid to heparan sulfate proteoglycans (HSPGs) expressed on the surface of hepatocytes [17], [20], [21]. Ad5 utilises the host FX protein, which circulates at approximately 8–10 µg/ml in the bloodstream, for cell binding since the cell surface interaction of the Ad5: FX complex is mediated through the serine protease domain of FX and not through a direct interaction of the virus with the cell surface [21]. This is of particular significance in the context of disseminated adenoviral disease affecting immunocompromised patients, since typing studies have found a predominance of species C adenoviruses in peripheral blood samples from these individuals [6], [22], [23]. Interestingly, recent surface plasmon resonance (SPR) studies have demonstrated that of 22 Ad species tested, from species A, B, C and D, 14 can bind FX [21] indicating that the interaction of Ad5 with FX may be highly conserved. Only adenoviruses from species D lacked the capacity to bind FX.

HSPGs are widely-expressed molecules composed of a core protein to which one or more heparan sulfate (HS) glycosaminoglycan (GAG) sidechains are covalently linked (reviewed in [24]). Their core protein diversity, structural heterogeneity and high negative charge (imparted by the HS-GAG sidechains, which consist of highly-sulfated disaccharide repeats of N-acetylglucosamine and glucuronic/iduronic acid) ensure that HSPGs play important roles in many biological processes [25]. Furthermore, several viral pathogens including the human immunodeficiency virus-1 (HIV) [26], human papilloma virus (HPV) [27], adeno-associated virus (AAV) [28] and herpes simplex virus (HSV) [29] exploit HSPGs as primary attachment receptors in different tissues and cell types. Although *in vitro* and *in vivo* studies suggest that the Ad5:FX complex interacts with membrane HSPGs [17], [30] the specific receptor requirements underlying FX-mediated adenoviral uptake have not been characterised. Interestingly, liver HS have been shown to possess a specialised structure, with much higher levels of *N*- and *O*-sulfation than HS from other tissues [31], [32], [33]. Viral interactions with HS sidechains at the cell surface are frequently associated with the presentation of a particular ‘sulfation signature’ [34], [35]. The substantial liver specificity of systemically disseminating Ad5 may therefore be due to the preferential interaction of Ad5:FX complexes with highly-sulfated liver HS.

Here, we investigated the receptor requirements for FX-mediated Ad5 cellular uptake *in vitro* and *in vivo*. We first showed in a number of cell lines that the interaction of the Ad5:FX complex with the cell surface was entirely independent of CAR and α_v_ integrins. By analysing Ad5 binding and uptake into enzymatically-pretreated cells we demonstrated that the primary attachment of the Ad5: FX complex was specifically mediated by HS sidechains. Detailed immunocytochemical analysis *in vitro* revealed delayed FX-mediated cell entry and cytosolic transport to the microtubule organising centre (MTOC) of a fluorescently-labelled Ad5 mutant lacking the penton base RGD motif, showing that rapid and efficient post-attachment kinetics is dependent on engagement of α_v_ integrins. Chemical inhibition or genetic ablation of endogenous HS sulfation completely abrogated FX-mediated Ad attachment and cell uptake, indicating that the Ad5: FX complex interacts with a specific HS sulfation pattern, while heparin-mediated inhibition of adenoviral gene transfer *in vitro* and Ad attachment to liver slices *ex vivo* was significantly attenuated in the absence of heparin *N*- and, in particular, *O*-linked sulfate groups. *In vivo*, Ad5 liver accumulation 1 h after intravenous administration to mice was significantly and dose-dependently inhibited by pre-inoculation with unmodified heparin but not by de-sulfated heparins. Immunohistochemical analysis of liver sections from mice intravenously injected with fluorescently-labelled Ad5 revealed localisation of Ad particles in CD31+ hepatic sinusoids and surrounding hepatocytes. We have thus integrated the established model of α_v_ integrin-dependent adenoviral infection with the FX-mediated pathway leading to liver uptake of Ad5.

## Results

### Heparan sulfate sidechains play an important role in FX-mediated Ad5 cell uptake *in vitro*

**Figure 1:**
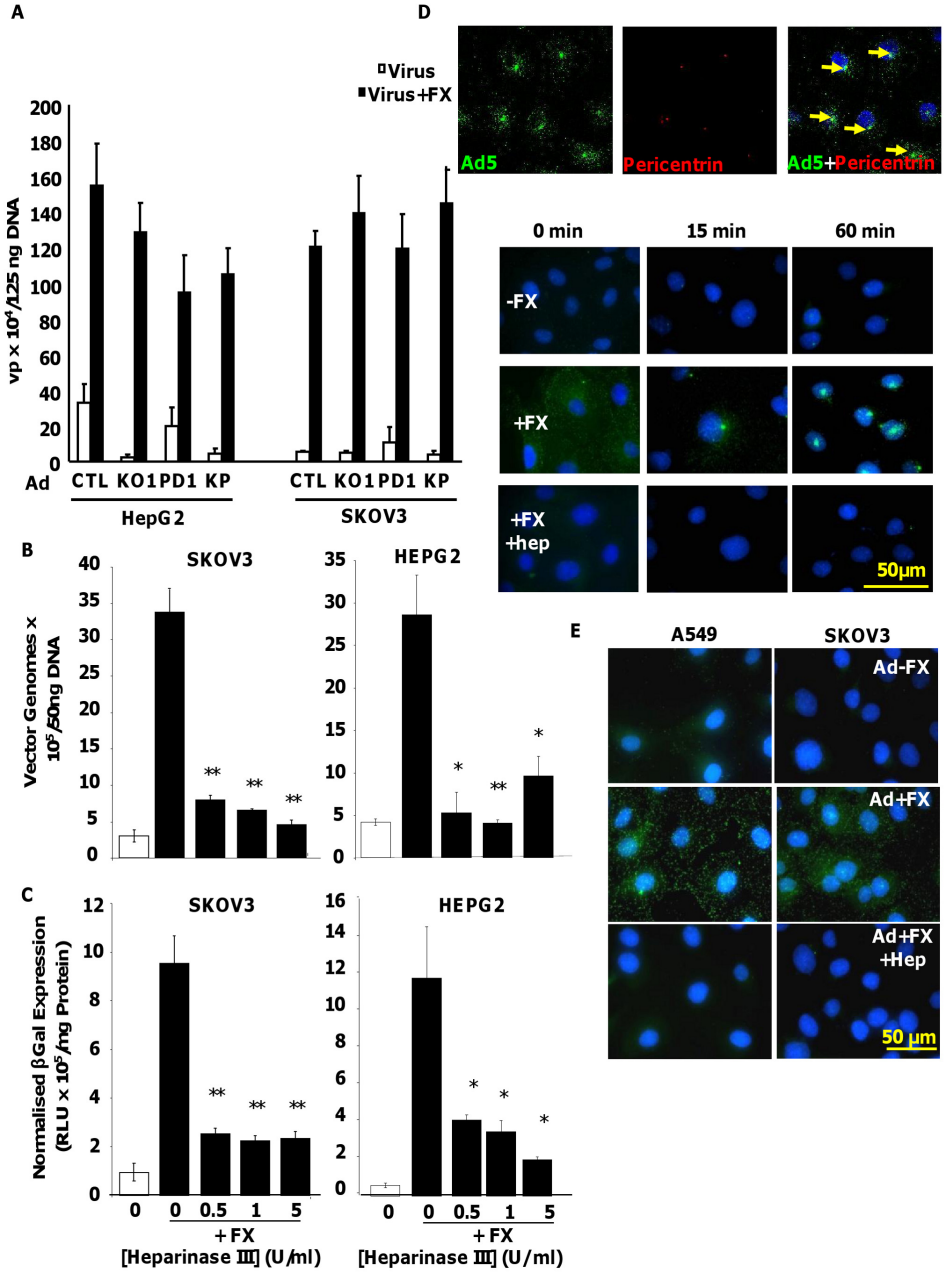
Importance of HS sidechains during Ad5 binding, cell entry, cytosolic transport and transduction *in vitro*. (**A**) Binding of 1000 vp/cell Ad5CTL, Ad5KO1(CAR-binding mutant), Ad5PD1 (α_v_ integrin-binding mutant) or Ad5KP(CAR- and α_v_ integrin-binding mutant) particles to cells was quantified after incubation of Ad particles with HepG2 or SKOV3 cells for 1 h at 4°C in the presence or absence of 10 µg/ml FX. Vector genomes were detected by quantitative PCR. (**B+C**) SKOV3 (left panels) or HepG2 (right panels) cells were incubated for 1 hour at 37°C in serum free media containing 0–5 U/ml of Heparinase III. The effect of heparinase pre-treatment on FX-mediated Ad5 binding (1000 vp/cell, 1 hour, 4°C) (B) or Ad5 mediated transduction (1000 vp/cell, 3 hours, 37°C) was quantified (C). ** = p<0.01. (**D**) **(upper panel)** 10,000 vp/cell of Alexa488-labelled Ad5 (green) were allowed to bind cells for 1 h at 4°C, then incubated at 37°C for 60 min prior to fixation and staining for the MTOC marker pericentrin (red). Nuclei were counterstained using DAPI. Colocalisation of fluorescently-labelled Ad5 particles with pericentrin is indicated by the yellow arrows. Images were captured on a confocal microscope under a 63× objective (**D**) **(lower panel)** Alexa-labelled Ad5 was allowed to bind cells for 1 h at 4°C in the presence or absence of 10 µg/ml FX and/or heparin (Hep). Cells were then incubated at 37°C for 0 min to 60 min prior to fixation. Nuclei were counterstained using DAPI. (**E**) Fluorescently-labelled Ad5 was allowed to bind cells for 1 h at 4°C in the presence or absence of 10 µg/ml FX and heparin (Hep). Nuclei were counterstained using DAPI. Images were captured under a 40× objective.

We utilised Ad5CTL (vector based on wild type Ad5 capsid), Ad5KO1 (CAR binding mutated), Ad5PD1 (integrin-binding mutant) or Ad5KP (both mutations) – see Methods for details. To assess the importance of HSPG heparan sulfate (HS) sidechains in Ad5 cell binding and uptake mediated by interaction with human FX we pre-treated human HepG2 hepatoma and SKOV3 ovarian carcinoma cells with heparinase III prior to performing Ad5 cell attachment and gene transfer experiments in the presence or absence of FX. Both HepG2 and SKOV3 cells express HSPGs, however unlike HepG2 cells, SKOV3 cells express very low levels of CAR [36]. Heparinase III is a heparin lyase that specifically cleaves N-acetylated (NAc) and transition domains of HS and can be used *in vitro* and *in vivo* to inhibit HS-mediated viral attachment [27], [37]. Cleavage of HS at NAc and transition domains was assayed by FACS. Heparinase III digestion significantly and dose-dependently reduced the percentage of cells positive for antibody 10E4, while significantly increasing the percentage of cells positive for antibody 3G10, confirming the substrate activity of heparinase III treatment (Fig. S1). To verify that heparinase III treatment had no effect on the widely-expressed GAG chondroitin sulfate, which is primarily composed of acetylgalactosamine hexosamine groups, cell surface chondroitin sulfate was quantified by FACS using the CS-56 mouse monoclonal antibody. While treatment of SKOV3 cells with chondroitinase ABC dose-dependently reduced the percentage of CS-56 positive cells, heparinase III treatment had no effect (Fig. S1), indicating that heparinase III treatment has no effect on chondroitin sulfate. Ad5 attachment to cells was substantially increased in the presence of FX in both HepG2 and SKOV3 cells (Fig. 1A and 1B; p<0.01). Similar results were observed when Ad5 mutants ablated for CAR-binding and/or α_v_ integrin binding (Ad5KO1, Ad5PD1 or Ad5KP; Fig. 1A) were used, in agreement with previous studies showing that the FX-mediated increase in virus cell attachment and gene transfer is CAR- and α_v_ integrin- independent [17], [30]. Similar FX-mediated enhancement of virus uptake was also observed in a panel of CAR^high^ and CAR^low^ cell lines when Ad5CTL and the α_v_ integrin-binding mutant Ad5PD1 were compared (Fig. S2). As expected levels of FX-mediated enhancement in gene transfer were lower in CAR^high^ cells compared to CAR^low^ cells (Fig. S1) since the CAR and FX pathways are both efficient *in vitro* pathways in the former. The FX-mediated increase in virus cell attachment was significantly attenuated by heparinase III pretreatment of HepG2 (p<0.01) or SKOV3 (p<0.05) cells (Fig. 1B). FX-mediated gene transfer was also significantly decreased after heparinase III pretreatment (Fig. 1C) of HepG2 and SKOV3 cells (p<0.01). We next carried out virus transport experiments *in vitro* using an Alexa488-labelled virus in the presence or absence of heparin. Heparin and HS sidechains have very similar structures, although heparin displays higher general sulfation than HS [25]. Heparin is therefore frequently used as a competitive inhibitor for HS binding [38], [39]. In the absence of heparin, Ad5:FX complexes were rapidly and efficiently transported to the MTOC in SKOV3 cells within an hour of adding fluorescently-tagged Ad5CTL:FX complexes to cells, as demonstrated by colocalisation with the MTOC marker pericentrin (Fig. 1D, upper panel). In the presence of heparin, however, FX-mediated attachment of fluorescently-tagged Ad5CTL to the cell surface was completely abrogated (Fig. 1D, lower panel), confirming that HS sidechains mediate Ad5CTL:FX attachment to the cell surface. Similar results were observed in SKOV3 and A549 human lung adenocarcinoma cells (Fig. 1E). Taken together, these results indicate that FX-mediated Ad5CTL cell attachment *in vitro* is dependent on the presence of HS sidechains.

### Optimal post-attachment cell entry and cytosolic transport of Ad5 is dependent on an intact penton base RGD motif

**Figure 2:**
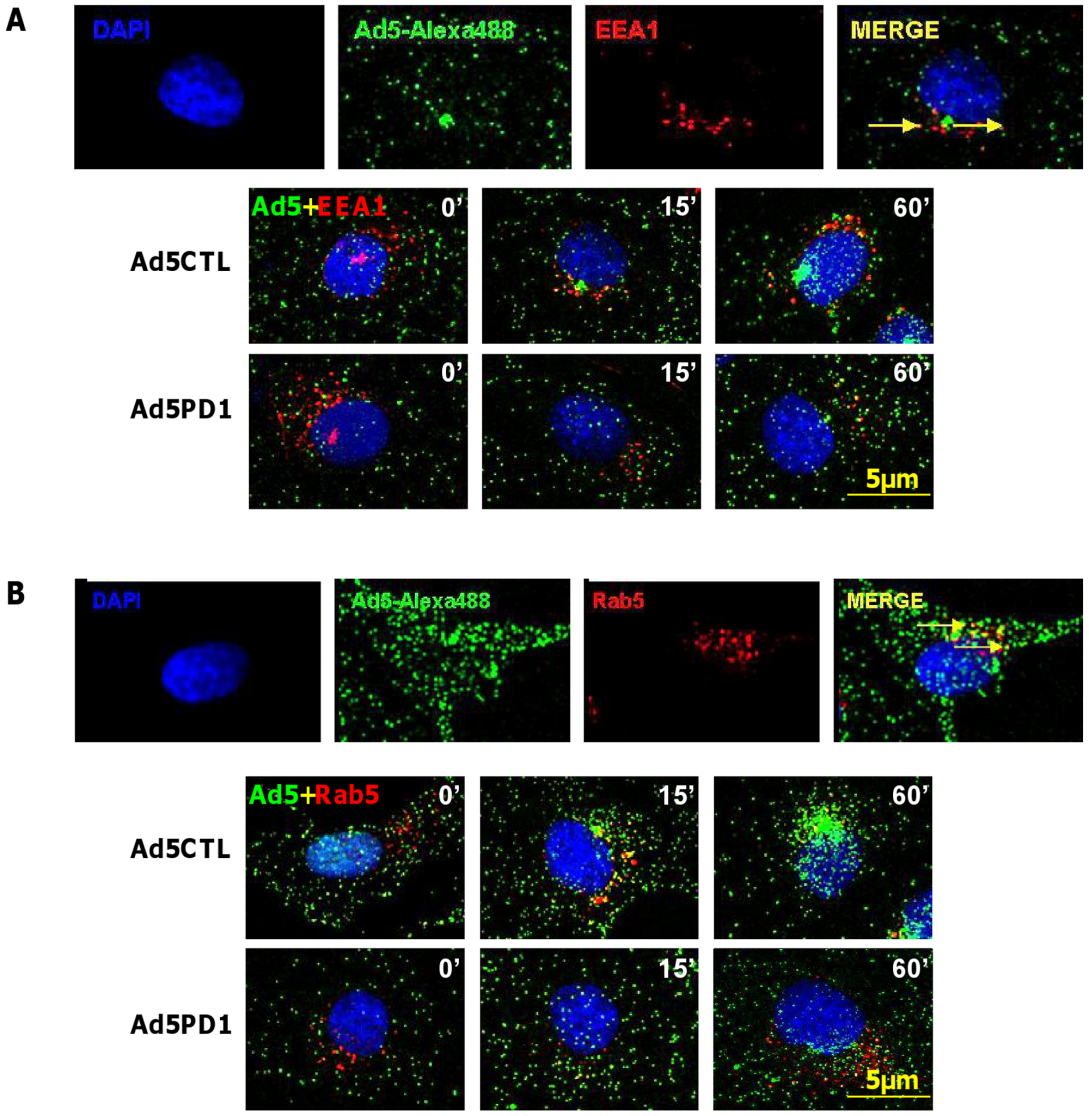
Endosomal localisation of αv integrin binding-defective Ad5 *in vitro*. (**A+B**) 10,000 vp/cell of Alexa488-labelled Ad5CTL or Ad5PD1 (green particles) in the presence of FX were allowed to bind cells for 1 h at 4°C, then incubated at 37°C for increasing lengths of time (15 min, 60 min) to allow internalisation and trafficking prior to fixation and staining for the early endosome markers EEA1 (**A**) or Rab5 (**B**). Specific binding of primary antibodies against EEA1 (**A, upper panel**) or Rab5 (**B, upper panel**) was detected using a Alexa546-labelled secondary antibody (red). Nuclei were counterstained using DAPI. Images were captured on a confocal microscope under a 63× objective.

**Figure 3:**
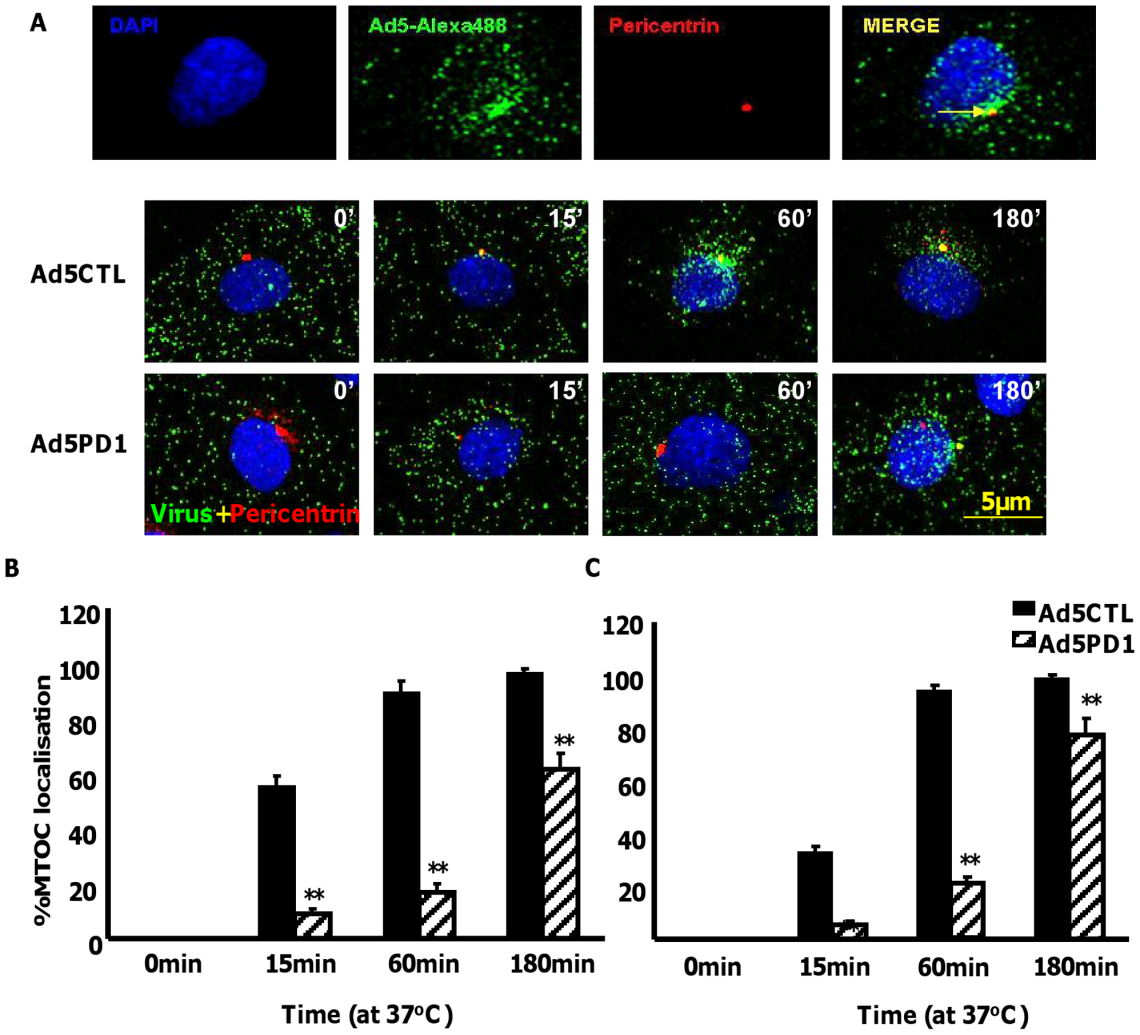
Delayed FX-mediated transport of αv integrin binding-defective Ad5 *in vitro*. 10,000 vp/cell of Alexa488-labelled Ad5CTL or Ad5PD1 (green particles) in the presence of FX were allowed to bind cells for 1 h at 4°C, then incubated at 37°C for increasing lengths of time (15 min, 60 min, 180 min) to allow internalisation and transport prior to fixation and staining for the MTOC marker pericentrin (**A**). Specific binding of a primary antibody against pericentrin (**A, upper panel**) was detected using an Alexa546-labelled secondary antibody (red). Nuclei were counterstained using DAPI. Images were captured on a confocal microscope under a 63× objective. (**B+C**) Percentage of cells with colocalisation of fluorescently-labelled Ad5CTL or Ad5PD1 with the MTOC marker pericentrin in SKOV3 (**B**) or A549 cells (**C**) was calculated by analysing at least 5 separate 40× microscope fields per experimental condition. ** = p<0.01 compared to Ad5CTL values, error bars represent S.E.M.

**Figure 4:**
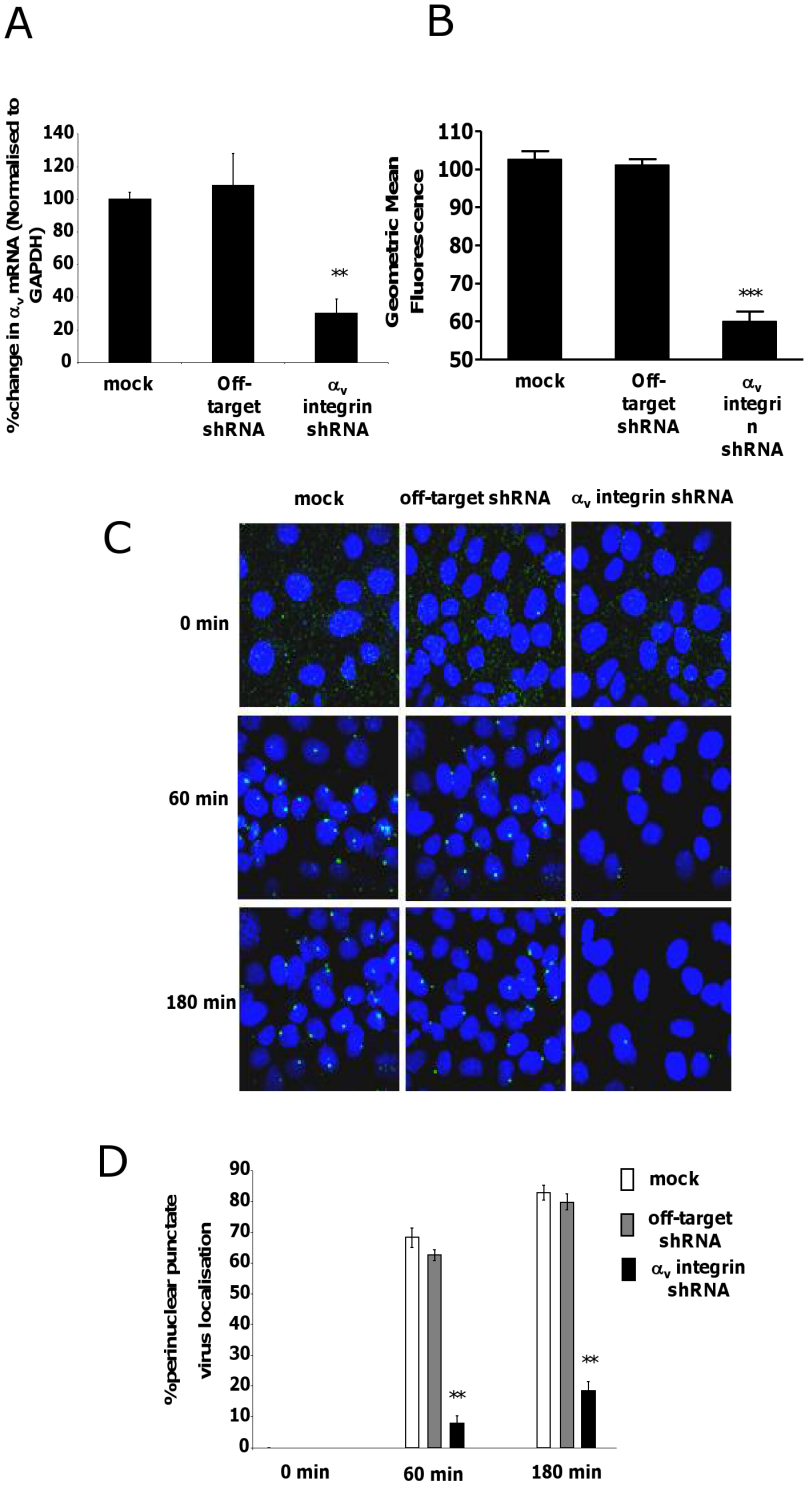
Effect of shRNA depletion of αv integrin on FX mediated Ad5 transport. Knockdown of αv integrin mRNA and protein expression levels in SKOV3 cells was quantified by qPCR (A) and flow cytometry (B), respectively. The effect of shRNA depletion on Ad5 trafficking in SKOV3 cells was assessed as previously. Representative images are shown (**B**) and quantification of peri-nuclear Ad5CTL localisation was calculated by analysis of 5 separate microscope fields per experimental condition (C). **p<0.05 vs both control conditions. (**D**) Percentage of SKOV3 cells with colocalisation of fluorescently-labelled Ad5CTL in the perinuclear region was calculated by analysing at least 5 separate 40× microscope fields per experimental condition. ** = p<0.01 compared to off target shRNA values, error bars represent S.E.M.

Although *in vitro* studies have shown that an intact penton base RGD motif is required for efficient endosome escape after CAR-mediated attachment of Ad5 to the cell surface [40] several *in vivo* studies indicate that ablation of the penton base RGD motif does not significantly affect liver uptake after systemic administration of Ad5 [14], [15], [16], [17], [41]. Having established that FX-mediated attachment of Ad5CTL to the cell surface requires HSPGs, we next assessed the role of α_v_ integrins during FX-mediated Ad5 cell uptake. *In vitro* cell tracking experiments were carried out in CAR^low^ SKOV3 [36] and CAR^high^ A549 cells [42] using Ad5 vectors with a mutated penton base RGD motif (Ad5PD1). Both Ad5CTL and Ad5PD1 efficiently bound the cell membrane in the presence of FX (Fig. 2A, B). In SKOV3 cells at 15 and 60 minutes post-internalisation, both Ad5CTL and Ad5PD1 particles had entered endosomal compartments as demonstrated by partial colocalisation with the early endosomal marker EEA1 and Rab5 (Fig. 2A, B). Similar results were observed in A549 cells (data not shown). MTOC colocalisation was quantified by assessing the proportion of cells in a 40× microscope field with colocalisation of fluorescently-labelled Ad5 particles (green) with the MTOC marker pericentrin (red; see upper panel in Fig. 3A, quantification Fig. 3B–C). Cell entry and cytosolic transport kinetics of the CAR binding-ablated vector Ad5KO1 closely resembled Ad5CTL, confirming that FX-mediated cell uptake does not require CAR (Fig. S3). Conversely, only 20–25% MTOC colocalisation was observed for Ad5PD1 in SKOV3 and A549 cells at the same timepoint (Fig. 3B and Fig. 3C, respectively). These data suggest that an integrin-mediated post-internalisation signal is required for optimal transport of Ad5CTL to the MTOC after FX-mediated cell surface attachment of Ad5CTL to HSPGs. To confirm the role of integrins we next performed a short hairpin (sh)RNA approach to knockdown α_v_ integrin expression in SKOV3 cells and assessed the effect of this on intracellular transport of Ad5CTL. Target knockdown was confirmed using TaqMan and flow cytometric analysis compared to mock-transfected and scrambled control shRNA cells (Fig. 4A, B). Knockdown led to a significant reduction in the localisation of Ad5CTL to the peri-nuclear compartment (Fig. 4B–C) thus confirming the importance of integrin engagement for transport via the FX-mediated pathway. We next determined the effect of kinase inhibitors that are known to affect cell entry and intracellular transport of adenovirus [43]. We used H89 dihydrochloride (an inhibitor of PKA), L Y294002 hydrochloride (an inhibitor of PI3K) and SB 203580 hydrochloride (an inhibitor of p38 MAPK). We observed that co-incubation of Ad5CTL-transduced cells in the presence of inhibitors of either PKA, PI3K or p38 MAPK was able to significantly reduce Ad5CTL-mediated transport to the MTOC in the presence of FX (Fig. S4). Since these molecules are linked to activation of cellular integrins during Ad internalisation or intracellular transport this further suggests that integrins are a component of the viral entry cycle in the presence of FX.

### Sulfation of HSPGs is critical for cell attachment and uptake of Ad5:FX

**Figure 5:**
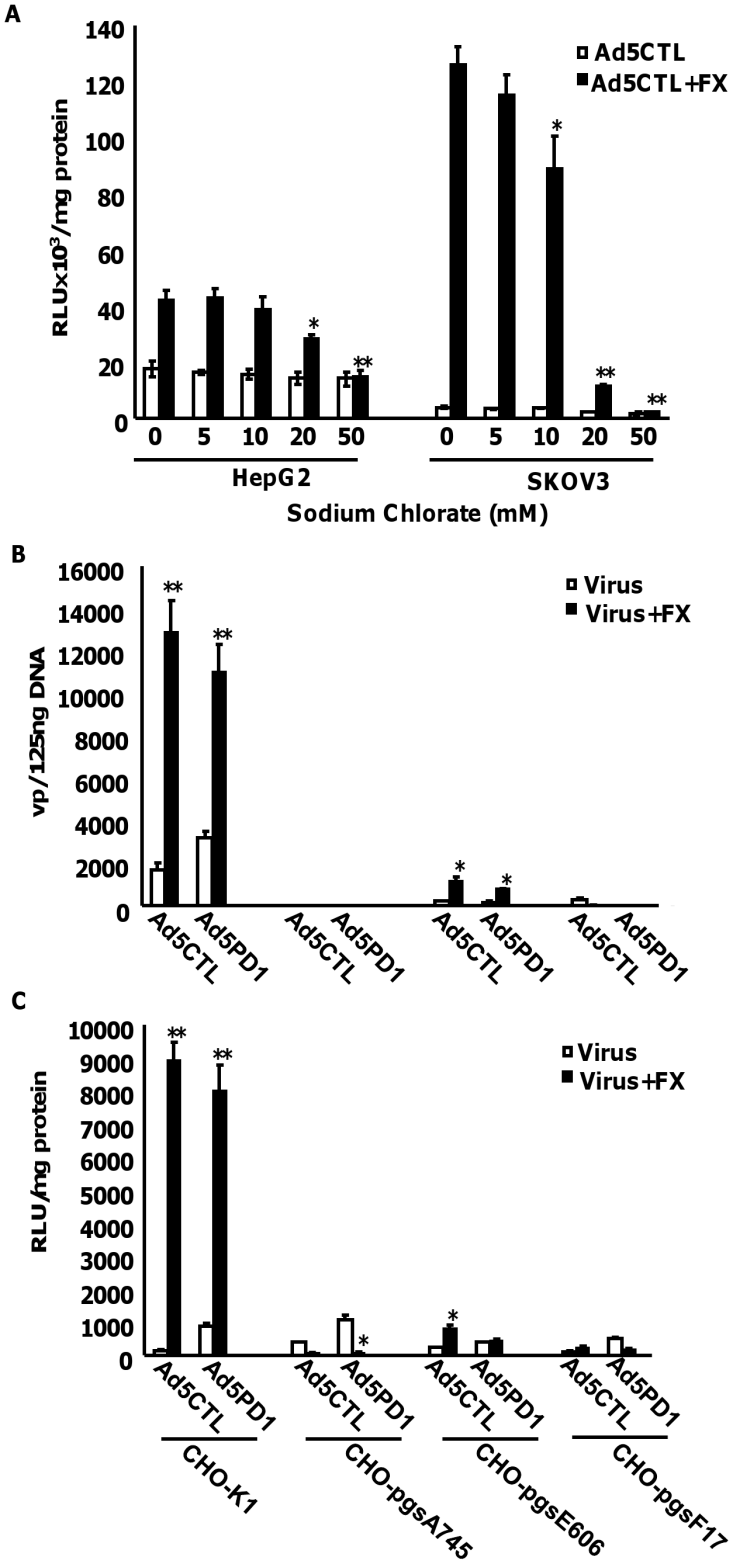
Importance of HSPG sulfation for FX-mediated Ad transduction *in vitro*. (**A**) HepG2 or SKOV3 cells that had been pretreated with increasing concentrations of the sulfation inhibitor sodium chlorate were transduced with 1000 vp/cell of Ad5CTL in the presence (closed bars) or absence (open bars) of 10 µg/ml FX. Pretreated cells were incubated with virus for 3 h at 37°C. Reporter gene activity was quantified 48 h post-transduction. * p<0.05, **p<0.01 compared to control values (0 mM). (**B+C**) Control CHO-K1, HS-deficient CHO-pgsA745, sulfation-low CHO-pgsE606 or 2-*O*-sulfation-deficient CHO-pgsF17 were transduced with 1000 vp/cell of Ad5CTL or Ad5PD1 in the presence or absence of 10 µg/ml FX. (**B**) Cell binding of Ad particles was quantified after incubation for 1 h at 4°C. Vector genomes were detected by quantitative PCR as described previously. (**C**) Ad5CTL or Ad5PD1 was incubated with cells for 3 h at 37°C. Reporter gene activity was quantified 48 hours post-infection. * p<0.05, **p<0.01 compared to -FX values.

Having established that FX-mediated Ad5CTL cell surface attachment required HS sidechains, we next investigated whether HS sidechain sulfation affects FX-mediated Ad5CTL cell uptake. *In vitro* experiments were therefore carried out in HepG2 and SKOV3 cells pretreated with increasing concentrations of sodium chlorate, a selective inhibitor of sulfation [44]. We confirmed that sodium chlorate treatment inhibited sulfation in a dose-dependent manner (Fig. S5A), reduced binding of Ad5CTL to cells in the presence of FX (Fig. S5B) in the absence of cellular toxicity (Fig. S5C). Gene transfer was used as a marker of successful cellular internalisation, cytosolic transport and nuclear uptake. FX-mediated Ad5CTL gene transfer was inhibited in a dose-dependent manner by pretreatment with sodium chlorate in both HepG2 and SKOV3 cells (Fig. 5A), suggesting that FX-mediated Ad5 uptake *in vitro* may depend on HS sidechain sulfation. No effect on basal levels of Ad5CTL uptake was observed. To assess the importance of HS sulfation for FX-mediated Ad5CTL transduction, cell attachment and transduction assays were carried out in CHO cell lines deficient in HS biosynthesis enzymes. Although FX induced a 60-fold increase in Ad5 cell attachment and uptake in parental CHO-K1 (CAR-) cells, no FX-mediated increase was observed in CHO-pgsA745 cells, which do not express xylosyltransferase-1 (XT1) and are consequently defective in HS-GAG synthesis [45] (Fig. 5B–C). The FX-mediated increase in Ad5 cell attachment and transduction was also significantly attenuated in N-deacetylase/N-sulfotransferase-1 (NDST1)-deficient CHO-pgsE606 cells, which synthesise HS chains with significantly reduced *O*- and, in particular, *N* sulfate groups [46] (Fig. 5B–C). Interestingly, FX was unable to increase Ad5CTL cell attachment and uptake in CHO-pgsF17 cells, which are 2-*O*-sulfotransferase-deficient and therefore lack 2-*O*-sulfated residues [47] (Fig. 5B–C). Similar results were obtained in all cell lines using the α_v_ integrin-binding mutant AdPD1 (Fig. 5B and 5C). Taken together, these data suggest that attachment of the Ad5CTL:FX complex to cell surface HSPGs *in vitro* requires HS sidechain sulfation.

### Removal of sulfate groups attenuates the ability of heparin to inhibit Ad5:FX binding and cellular uptake *in vitro* and *ex vivo*

To confirm the importance of HS sidechain sulfation for FX-mediated Ad5CTL cell attachment and uptake, *in vitro* gene transfer and *ex vivo* attachment experiments were carried out in the presence of heparan sulfates or heparins with biosynthetically-modified sulfation. Bovine intestinal heparin and porcine intestinal heparan sulfate are very highly-sulfated [39]. In contrast, porcine kidney heparan sulfate possesses fewer *N*- and *O*- sulfate groups, while de-*N*-sulfated heparin lacks *N*-sulfated glucosamine residues [39] and de-*O*-sulfated heparin lacks *O*-sulfate groups [39], [48]. Competitive inhibition experiments *in vitro* and *ex vivo* were carried out in SKOV3 cells or mouse liver slices, respectively, in the presence or absence of FX.

**Figure 6:**
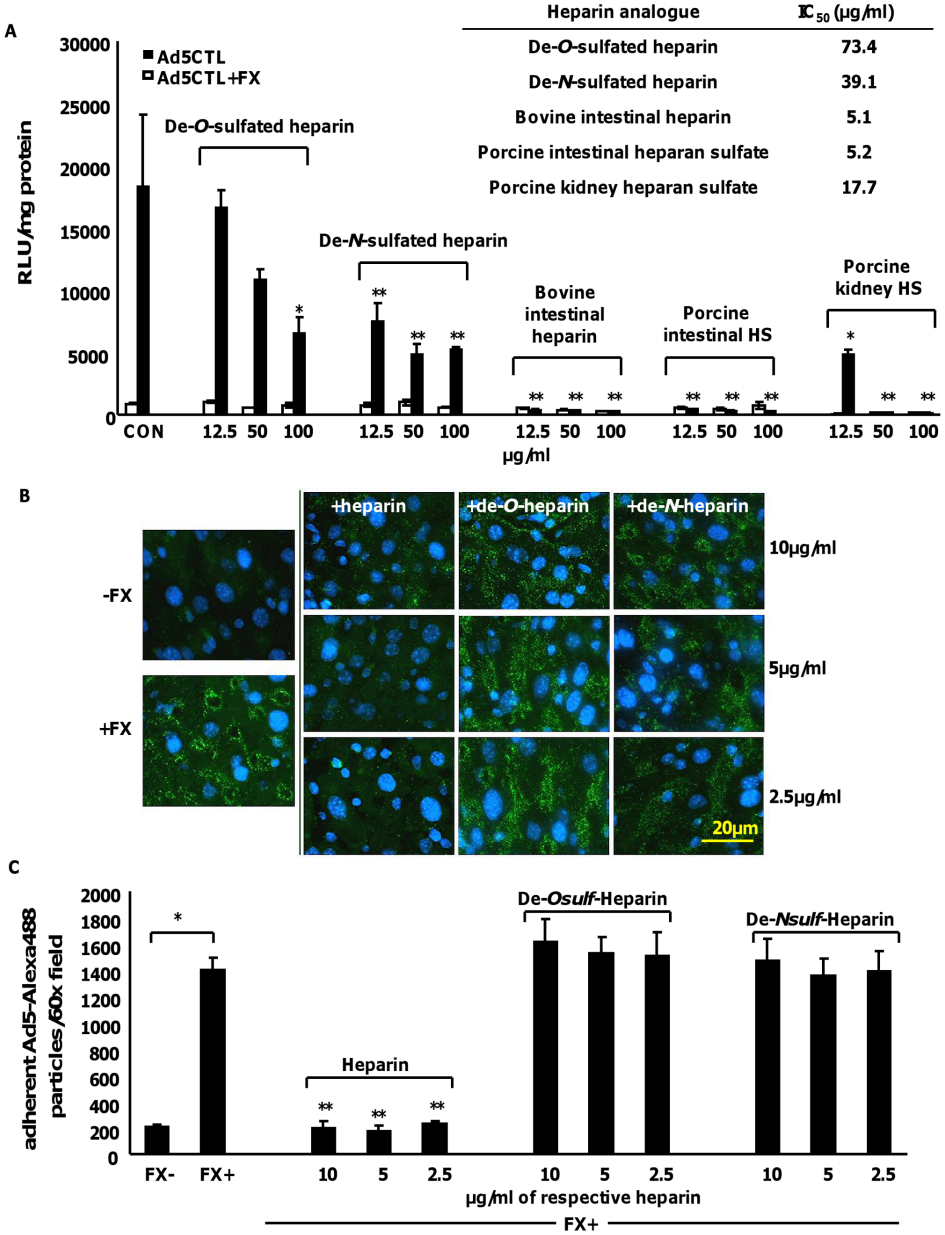
The inhibitory effects of heparin on FX-Ad uptake *in vitro* and *ex vivo* are sulfation-dependent. (**A**) SKOV3 cells were transduced with 1000 vp/cell of Ad5 in the presence or absence of 10 µg/ml FX and varying concentrations of heparins/heparan sulfates for 3 h at 37°C. Reporter gene expression was quantified 48 h post-transduction. IC_50_ (µg/ml, inset) values were calculated using the Hill-Slope model. *p<0.05, **p<0.01 compared to CON+FX (i.e. no heparin) values. (**B**) Liver slices from MF1 mice were incubated with 1×10^8^ vp of Alexa488-labelled Ad5CTL (green) in the presence or absence of 10 µg/ml FX and increasing concentrations of heparins for 1 h at 4°C. Nuclei were counterstained using DAPI. Images were captured using a 60× microscope objective. (**C**) Attachment of Alexa488-labelled Ad5CTL particles to mouse liver slices were quantitated using the automated cell counting function in ImageJ. Data represent the average number of particles per 60× microscope field. ^#^p<0.01 vs FX− conditions, ** = p<0.01 vs FX+ conditions in the absence of heparin.

Dose-dependent inhibition of FX-enhanced Ad5CTL gene transfer into SKOV3 cells was observed in the presence of all heparins and heparan sulfates (Fig. 6A). However the IC_50_ values for the highly-sulfated bovine intestinal heparin and porcine intestinal heparan sulfate (5.1 µg/ml and 5.2 µg/ml respectively) were lower than the IC_50_ values for the de-sulfated heparins (39.1 µg/ml and 73.4 µg/ml for de-*N*-sulfated or de-*O*-sulfated heparins respectively) or heparan sulfate with reduced sulfation (porcine kidney heparan sulfate, 17.7 µg/ml) (Fig. 6A). Similar results were obtained when CAR- or α_v_ integrin binding-ablated viruses were used (Table S1).

Next, the attachment of fluorescently-labelled Ad5CTL (green) to mouse liver slices *ex vivo* was analysed. Adherent Ad5CTL particles were quantified by analysing captured images of 60× microscope fields using the ImageJ automated cell counting function. Co-incubation with FX significantly increased the attachment of fluorescently-labelled Ad5CTL to liver sections (Fig. 6B and Fig. 5C). At the concentrations used, heparin significantly inhibited FX-mediated attachment of Ad5CTL to liver sections (p<0.01; Fig. 6B–C). However neither de-*O*-sulfated nor de-*N*-sulfated heparin inhibited the attachment of Ad5CTL to liver sections in the presence of FX. These results indicate that binding of the Ad5CTL-FX complex to heparin/HS *in vitro* requires the presence of highly anionic sulfate groups, supporting the hypothesis that FX-mediated Ad5CTL cell uptake is dependent on HS sidechain sulfation.

### Inhibition of Ad liver accumulation *in vivo* by heparin is sulfation-dependent

To identify whether the sulfation status of hepatocyte HS contributes to the liver uptake of Ad5CTL from the circulation, competitive inhibition experiments were carried out *in vivo* in the presence of heparins with biosynthetically-modified sulfation. Mice were inoculated with increasing concentrations of heparins prior to intravascular administration of Ad5CTL. Ad5CTL liver uptake at an early timepoint post-inoculation was then quantified by assessing viral genome accumulation 1 h post-administration. Cellular localisation of Ad5CTL in mouse livers was analysed by staining liver sections with CD31 to visualise the vasculature in conjunction with fluorescently labelled Ad5CTL. We have previously shown that the FX-pathway is hepatocyte-specific and Kupffer cell uptake is unaffected by pharmacological modulation or genetic approaches to modify FX binding as Kupffer cell uptake is a scavenging process [49], [50], [51], [52]. We therefore used macrophage-depleted mice to allow selective visualisation of HSPG uptake mechanisms via the FX pathway *in vivo* at an early time point post injection.

**Figure 7:**
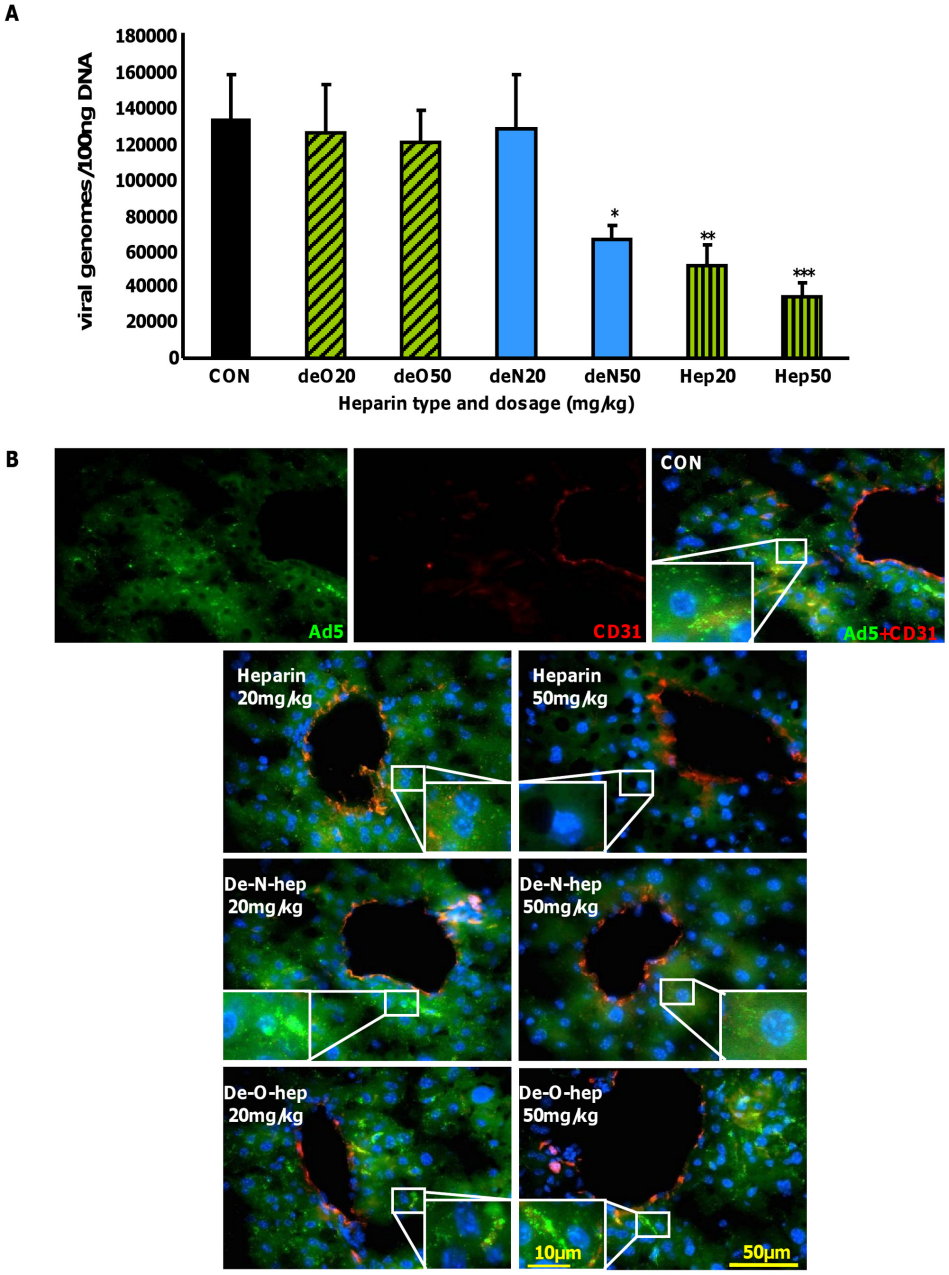
Effect of modified heparins on Ad5 liver accumulation. (**A**) Heparin or modified heparins were administered to macrophage-depleted MF1 mice 5 minutes prior to intravenous injection of 1×10^11^ vp of Ad5. Mice were sacrificed 1 h later and Ad genomes were detected in liver tissue lysates by quantitative PCR as previously described (n = 7). *p<0.05, **p<0.01. (**B**) Control (no heparin), heparin or modified heparins were administered to macrophage-depleted MF1 outbred mice 5 minutes prior to intravenous injection of 1×10^11^ vp of fluorescently-labelled Ad5 (green). Mice were sacrificed 1 h later and livers were fixed then stained for the endothelial marker CD31 to facilitate imaging of the sinusoids in the liver (red). Nuclei were counterstained using DAPI. Images were captured using a 40× microscope objective.

Heparin pre-inoculation at both 20 mg/kg and 50 mg/kg significantly and dose-dependently inhibited Ad5CTL accumulation in the liver 1 h post-inoculation (p<0.01)(Fig. 7A). Although no effect was observed at either dose of de-*O*-sulfated heparin (20 mg/kg or 50 mg/kg), significantly fewer Ad5CTL genomes were detected in livers of mice pre-treated with 50 mg/kg de-*N*-sulfated heparin (*p*<0.05)(Fig. 7A). Immunofluorescence staining for CD31 was performed to facilitate identification of endothelial sinusoids in the liver architecture. Sections from control mice inoculated with fluorescently-labelled Ad5CTL (green) showed accumulation of Ad5CTL particles in liver sinusoids and on the surface of hepatocytes (Fig. 7B, upper panel). Administration of heparin (both 20 mg/kg and 50 mg/kg doses) or high-dose de-*N*-sulfated heparin clearly reduced accumulation of labelled Ad5 while de-*O*-sulfated heparin had no effect at either dose (Fig. 7B, lower panel).

Taken together, our data indicate that the hepatic uptake observed after intravenous administration of Ad5CTL is dependent on HS sidechain sulfation, with a particular requirement for *O*-sulfation. In conjunction with previous studies showing that liver HS is highly enriched in *2-O*-sulfated residues [31] our *in vitro*, *ex vivo* and *in vivo* results further suggest that the hepatic tropism of Ad5CTL may be due to preferential binding of the Ad5:FX complex to HS sidechains.

## Discussion

Previous studies have shown that the liver uptake of Ad5 after exposure to the circulation is dependent on FX binding directly to the hexon protein in the Ad5 capsid, putatively via a FX-mediated interaction with hepatocyte membrane HSPGs [17], [30]. In the present study we investigated the functional receptor requirements for the Ad5CTL:FX complex using a variety of *in vitro*, *ex vivo* and *in vivo* experimental approaches.

We have demonstrated that FX-mediated Ad5CTL cell attachment *in vitro* requires the presence of HS sidechains but not CAR or α_v_ integrins. FX-mediated Ad5CTL binding to CAR^high^ HepG2 and CAR^low^ SKOV3 cells was not affected by fiber knob or penton base mutations ablating CAR- or α_v_ integrin-interacting motifs, respectively, indicating that neither CAR nor α_v_ integrins were required for the FX-mediated primary interaction with the cell surface. Conversely, cleavage of HS sidechains by heparinase III pretreatment significantly inhibited FX-mediated Ad5 attachment to and uptake into HepG2 and SKOV3 cells. Heparin, a highly-sulfated HS analogue, also abrogated FX-mediated adenoviral cell surface binding. Moreover, no FX-mediated enhancement of Ad5CTL cell binding or gene transfer was observed in CHO-pgsA745 cells which do not display HS sidechains. Taken together, these results clearly demonstrate that the primary interaction of the Ad5CTL:FX complex with the cell surface is mediated via HS sidechains. Although our data indicate that CAR- or α_v_ integrins are not required for FX-mediated attachment of Ad5CTL to the cell surface, intracellular transport experiments using a mutant with an ablated penton base RGD motif (Ad5PD1) and a knockdown shRNA approach revealed that efficient and rapid post-internalisation transport of virus particles to the nucleus requires engagement of RGD-interacting integrins. A similar delay in intracellular transport of Ad5PD1 was observed in CAR^high^ A549 and CAR^low^ SKOV3 cells, suggesting that the altered transport was not affected by differences in CAR expression. Interestingly, a previous study investigating the cytoplasmic transport of Ad5 after CAR-mediated cell surface attachment has demonstrated a similar reliance on RGD-integrin interactions [40]. In conjunction with this study, our results indicate that integrin engagement is required for rapid and efficient intracellular transport of Ad5CTL regardless of the primary attachment receptor used. Furthermore, the potential HS sidechain dependence of FX-mediated cell surface attachment suggests that the Ad5CTL:FX complex may utilise HSPGs as attachment factors in a similar manner to other hepatotropic viruses such as HSV [53] hepatitis [34], [54] and AAV-2 [55], [56].

A central aim of the present study was to establish whether FX-mediated Ad5CTL cell uptake is dependent on the degree or type of HS sidechain sulfation. Blocking HS sulfation by pre-incubating cells with increasing concentrations of sodium chlorate dose-dependently inhibited FX-mediated Ad5CTL gene transfer in HepG2 and SKOV3 cells. Moreover, FX-mediated enhancement of Ad5CTL cell attachment and uptake was significantly attenuated in CHO-pgsE606 cells, which have reduced overall sulfation due to a deficiency in the *N*-deacetylase/*N*-sulfotransferase-1(NDST1) gene [46]. In addition, IC_50_ values for porcine kidney heparan sulfate, which possesses fewer sulfate groups than heparin, were approximately 3-fold higher than IC_50_ values for highly-sulfated bovine intestinal heparin or porcine intestinal heparan sulfate. These data show that FX-mediated Ad5 cell attachment and uptake is dependent on the degree of HS sidechain sulfation. Interestingly, removal of *N*- or *O*- sulfate groups significantly attenuated the inhibitory capabilities of heparin on Ad5CTL uptake *in vitro*, increasing IC_50_ values approximately 8- or 14-fold respectively. Furthermore, unlike native heparin, de-*O*-sulfated and de-*N*-sulfated heparins were unable to inhibit FX-mediated attachment of fluorescently-labelled Ad5CTL to liver slices *ex vivo*. Finally, no FX-mediated enhancement of Ad5CTL cell attachment or uptake was observed in CHO-pgsF17 cells, which lack 2-*O*-sulfate groups due to a deficiency in the 2-*O*-sulfotransferase gene [47]. Taken together, our results suggest that while the degree of sulfation modulates the FX-mediated uptake of Ad5CTL *in vitro*, the Ad5CTL:FX complex may also preferentially interact with specific sulfate moieties.

A number of previous studies have examined the biochemical composition of heparan sulfate from different tissues and have shown that liver heparan sulfate is enriched in sulfated moieties, in particular 2-*O* sulfate groups [31], [33]. Interestingly, hepatic clearance of intravenously-administered very low density lipoprotein (VLDL) is significantly reduced in mice with liver-specific knockout of the heparan sulfate 2-*O* sulfotransferase enzyme [57], thereby adding mechanistic insight into previously published work documenting increased levels of systemic VLDL in mice with reduced overall liver HS sulfation [32]. These studies show that the specialised structure of liver HS can contribute to the hepatic accumulation and uptake of circulating particles, and indicate how liver HS sulfation may contribute to the accumulation of systemically-disseminated, FX-interacting adenoviruses such as Ad5. The final aim of this study was therefore to investigate the importance of HS sidechain sulfation in FX-mediated Ad5CTL interactions with liver cells *in vivo*. While pre-injection of native heparin or high-dose de-*N*-sulfated heparin significantly attenuated Ad5CTL genome accumulation in the livers of macrophage-depleted mice 1 h after intravenous delivery, administration of de-*O*-sulfated or low-dose de-*N*-sulfated heparin had no effect. Immunohistochemical analysis of fluorescently-labelled Ad5CTL in liver sections from these mice clearly showed a significant reduction in Ad5CTL accumulation around CD31+ hepatic sinusoids after pre-injection of high-dose native and de-*N*-sulfated heparin, but not de-*O*-sulfated heparin. These results are consistent with our *in vitro* and *ex vivo* data, suggesting that HS sidechain sulfation (in particular *O*-sulfation) may contribute to the accumulation of Ad5CTL in the liver at this timepoint after intravenous administration. Taken together, our data indicate that the FX-mediated interaction of Ad5CTL with HSPGs both *in vitro* and after intravascular administration *in vivo* involves the presentation of a ‘sulfation signature’.

The domains of FX responsible for mediating Ad5 transduction of hepatocytes have been demonstrated. The Gla domain of FX docks in the cup at the centre of each hexon trimer and the virus:FX complex is then delivered to the hepatocyte surface via a heparin binding exosite in the FX serine protease domain which tethers to HSPGs at the cell surface [21]. While activated FX (FXa) has previously been shown to bind to the cell surface of hepatocytes and tumour cell lines, this interaction was not observed with FX [58]. The cell surface receptors that mediate FXa interactions with hepatocytes were later shown to be tissue factor pathway inhibitor and nexin-1 and required a functional FXa active site [59]. Previously, it was also shown that a Ca^2+^-mediated interaction between the Gla domain of FX and phospholipid components of the cell membrane mediated cell surface interactions [60], [61]. A similar phospholipid-mediated interaction between FX and platelets has also been reported [62]. FX has also been previously shown to mediate interactions with the cell membrane of other cell types via other identified receptors. For example, in whole human blood FX has been shown to bind monocytes via the α_M_β_2_ integrin (CD11b/CD18), resulting in its activation via cathepsin G-mediated cleavage, although the domain of FX that binds CD11b was not identified [63].

Previous studies have shown that binding of Ad3 fiber knob to HSPGs *in vitro* is also dependent on HS sidechain sulfation [64]. A putative HSPG-binding region has been identified in the Ad5 fiber shaft (_91_KKTK_94_) [41]. However while reduced hepatic uptake was observed after intravascular administration of a virus harbouring a mutation of the KKTK motif (_91_KKTK_94_→GAGA), *in vitro* assays showed that this virus was deficient in intracellular transport [65]. A recent study has clearly demonstrated that fiber is not involved in binding of the Ad5:FX complex to the cell surface, since a fiberless Ad5 mutant showed no significant reduction in FX-mediated cell surface attachment [21]. This study also showed that the Gla domain of FX binds to hypervariable regions in the Ad5 hexon [21], while positively-charged residues in the FX serine protease domain putatively interact with HSPGs [21], [66], [67], [68]. It is therefore likely that the HS sidechain-dependent interaction of Ad5 with the cell surface is mediated by FX ‘bridging’ to hexon capsid proteins rather than by a direct interaction with fiber.

In the past two decades there has been significant interest in the potential use of sulfated polysaccharides such as heparin and heparan sulfates in antiviral therapy (reviewed in [69]). For example, the polyanionic compound PRO 2000 competitively inhibits attachment of the HIV-1 envelope protein gp120 to HSPGs on CD4+ T cells and is currently under development as a topical antiviral gel to prevent cervical HIV-1 transmission [70], [71]. However undesirable side-effects such as anticoagulation limit the use of certain highly-sulfated, high molecular weight polysaccharides, including heparin. As stated previously, viral interactions with HS sidechains at the cell surface are often associated with the presentation of a particular ‘sulfation signature’. For instance, hepatitis E cell binding is thought to be dependent on 6-*O* sulfation [34] while the interaction of HSV-1 with the surface of target cells during infection *in vivo* is mediated by 3-*O* sulfate moieties [35]. Knowledge of the specific positioning and number of sulfate groups required for optimal virucidal activity has facilitated the development of targeted antiviral polyanions such as carrageenan and cellulose sulfate, which have significantly fewer side-effects [69]. This underlines the therapeutic relevance of fully understanding the sulfation requirements for Ad5:FX attachment to host cell HSPGs. This is of particular importance in the context of disseminated adenoviral disease in immunocompromised patients, as several studies have identified FX-binding species C adenoviruses in peripheral blood samples from these individuals [6], [7], [8], [9]. Further detailed studies on the receptor-mediated interactions of Ad5 in circulation are now required to fully characterise the factors underlying the clinical pathogenicity of this virus.

## Materials and Methods

### Ethics statement

All animal experiments were approved by the University of Glasgow Animal Procedures and Ethics Committee and performed under UK Home Office licence (PPL 60/3752) in strict accordance with UK Home Office guidelines.

### Materials

Purified human blood coagulation factor X (FX) was purchased from Cambridge Biosciences (Cambridge, UK). Heparinase III, chondroitinase ABC, bovine intestinal heparin, porcine intestinal heparan sulfate, porcine kidney heparan sulfate, de-N-sulfated heparin, de-O-sulfated heparin and sodium chlorate were obtained from Sigma (Sigma-Aldrich, Gillingham, UK). Primary antibodies raised against EEA1, Rab5, pericentrin or α-tubulin were obtained from Abcam (Cambridge, UK). Primary antibodies raised against intact heparan sulfate (clone 10E4) or heparinase III-digested heparan sulphate (clone 3G10) were obtained from AMS Biotechnology (Oxford, UK). The primary antibody raised against chondroitin sulfate (clone CS-56) was obtained from Sigma (Sigma-Aldrich, Gillingham, UK). All secondary antibodies were obtained from Invitrogen (Paisley, Scotland, UK). The kinase inhibitors LY 294002 hydrochloride, H 89 hydrochloride and SB 202190 hydrochloride were obtained from Tocris Bioscience (Bristol, UK).

### Cell lines, cell culture and virus production

A549 (human lung carcinoma ATCC CCL-185), HT29 (human colorectal adenocarcinoma: ATCC HTB-38), MDA-MB-231 (human breast adenocarcinoma: ATCC HTB-26) NCI-H522 (human lung adenocarcinoma: ATCC CRL-5810), SKOV3 (human ovarian carcinoma: ATCC HTB-77) and SNB19 cells (human glioblastoma: ATCC CRL-2219) were grown in RPMI 1640 medium supplemented with 10% fetal calf serum, 2 mM L-glutamine and 1% penicillin-streptomycin (Invitrogen, Paisley, UK). HepG2 (human hepatocellular carcinoma: ATCC CRL-11997) and 293 (Human Embryonic Kidney: ATCC CRL-1573) cells were grown in Dulbecco's Modified Eagle's Medium (DMEM; Invitrogen, Paisley, UK) supplemented with 10% fetal calf serum, 2 mM L-glutamine and 1% penicillin-streptomycin. CHO-pgsA745 (ATCC: CRL-2242), CHO-pgsE606 (ATCC: CRL-2246) and CHO-pgsF17 cells [47] were grown in Ham's F-12 medium (Invitrogen, Paisley, UK) supplemented with 10% fetal calf serum, 2 mM L-glutamine and 1% penicillin-streptomycin. The E1/E3-deleted Ad5CTL adenovirus encodes a Rous sarcoma virus (RSV) promoter-driven *LacZ* expression cassette as described previously [66]. Ad5KO1 is based on Ad5CTL and contains a two-amino acid substitution in the fiber knob (S408E, P409A) that ablates CAR binding. Ad5PD1 is also based on Ad5 and contains a substitution of amino acids 337–344 of the penton base gene (HAIRGDTF) with amino acids SRGYPYDVPDYAGTS, ablating RGD-mediated α_v_ integrin-binding. Ad5KP contains both fiber knob (KO1) and penton base (PD1) mutations. Viruses were propagated in 293 cells and purified by CsCl gradient centrifugation. Vector genomes were quantified by SYBR green quantitative polymerase reaction (qPCR) on an Applied Biosystems ABI Prism 7700 sequence detection system using primers directed against the LacZ transgene (forward 5′-ATCTGACCACCAGCGAAATGG-3′ and reverse 5′-CATCAGCAGGTGTATCTGCCG-3′). Viral particles were determined by micro bicinchoninic-acid assay (Perbio Science, Cramlington, UK) using the formula 1 µg protein = 4×10^9^ VP [72].

### Virus labelling

Adenoviruses were fluorescently labelled using an Alexa Fluor-488 (green) protein labelling kit according to the manufacturer's instructions (Invitrogen, Paisley, UK). Free label was dialysed from labelled Ad5 using 10,000 molecular weight cut off slide-a-lyzer cassettes (Perbio Science, Cramlington, UK) overnight in 100 mM Tris, 50 mM EDTA. A dye: virus particle ratio of 3∶1 was used for all labelling reactions. Fluorescent dye labelling efficiency was assessed using the ‘proteins and labels’ function on a Nanodrop-1000 spectrophotometer. Infectivity of labelled adenoviruses was verified by *in vitro* gene transfer assay as described below.

### Flow cytometric analysis of heparinase III pretreatment

FACS was performed on SKOV3 cells cultured under the conditions described above. Cells were detached from culture vessels using a 1× citric saline solution and counted using trypan blue exclusion. Cells were then resuspended in serum-free DMEM (SFDMEM) at a concentration of 4×10^6^ cells/ml. Heparinase III or chondroitinase ABC was then added to 50 µl of this cell suspension at the required concentrations (0 U/ml, 0.5 U/ml, 1 U/ml, 5 U/ml) and incubated with cells in a shaking waterbath at 37°C for 1 h. Cells were washed twice in SFDMEM) then incubated with primary antibodies (10E4, 3G10 or CS-56; all mouse monoclonal antibodies) or a matching isotype control in SFDMEM containing 0.1% BSA for 30 min on ice. Cells were then washed twice and incubated with a FITC-labelled secondary antibody in SFDMEM for 30 minutes on ice. Cells were washed twice and cell labelling was then detected on a FACS Canto II flow cytometer (Beckton Dickinson, Oxford, UK) using FACS DIVA software. Viable cells were gated by their FSC/SSC profiles, with a minimum of 5000 gated events analysed per sample. Results are expressed as percentage positively-stained cells per sample, from 3 independent samples analysed in triplicate.

### Measurement of sulfated glycosaminoglycan (sGAG) content

Total sulfated glycan content was measured in cell lysates using the Blyscan sulfated GAG assay kit (Biocolour, Newtonabbey, Northern Ireland, UK) according to manufacturer's instructions. Briefly, cultured cells were lysed in RIPA buffer (50 mM Tris, 150 mM NaCl, 0.1% SDS, 0.5% sodium deoxycholate, 1% NP40) then lysates were incubated with a molar excess of the cationic, sulfate-binding dye 1,9 dimethylmethylene blue. Lysates were pelleted and unbound dye was removed. Soluble GAG content was measured by determining the quantity of bound dye by spectrophotometric standard curve analysis at 656 nm. Protein concentrations were measured by Bicinchoninic Acid Assay (Perbio Science, Cramlington, UK) as described above. Data are expressed as µg sGAG/mg protein.

### Analysis of Ad5 transport *in vitro*

Cells were seeded in 4-well chamber slides at 1×10^5^ cells/well 24 h prior to assay. Cells were gently washed with PBS then incubated with 1×10^4^ vp/cell in 300 µl SFDMEM for 1 h on ice. Factor X and bovine intestinal heparin were both used at a concentration of 10 µg/ml. Cells were then gently washed with PBS and incubated at 37°C for 15, 30, 60 or 180 minutes prior to fixation. Localisation of Ad particles at the MTOC was characterised by staining cells using a polyclonal rabbit pericentrin antibody (1∶200 dilution: Abcam, Cambridge, UK) while localisation of Ad particles in early endosomes was characterised by staining cells using a polyclonal rabbit EEA1 antibody (Early Endosome Antigen-1) or a polyclonal rabbit Rab5 antibody at a 1∶200 dilution (Abcam, Cambridge, UK). Cell morphology was assessed using a polyclonal mouse α-tubulin antibody at a 1∶500 dilution (Abcam, Cambridge, UK). Specific binding of primary antibodies was visualised using a goat anti-rat Alexa Fluor 546 (red) secondary antibody in PBS at a dilution of 1∶500. Cells were imaged using a Zeiss confocal imaging system (LSM500). Colocalisation of Ad5 with the MTOC was quantified by visually assessing the percentage of cells with Alexa488-virus and pericentrin co-staining. Data were averaged from 5 40× microscope fields per experimental condition.

### Analysis of Ad5 trafficking in vitro in the presence of kinase inhibitors

Cells were seeded in 8-well chamber slides at 5×10^4^ cells/well 24 h prior to assay. Cells were gently washed with PBS then incubated with 100 µM LY 294002 hydrochloride, 40 µM H 89 hydrochloride or 10 µM SB 202190 hydrochloride (Tocris Bioscience, UK) for 30 minutes at 37°C. Cells were gently washed with PBS then incubated with 1×10^4^ vp/cell of Alexa Fluor-488 labelled virus in the presence of 100 µM LY 294002 hydrochloride, 40 µM H 89 hydrochloride or 10 µM SB 202190 hydrochloride in 150 µl SFDMEM for 1 h on ice. Factor X was used at a concentration of 10 µg/ml. Cells were incubated at 37°C for 180 minutes prior to fixation. Localisation of Ad particles at the MTOC was characterised and quantified as previously described.

### Analysis of shRNA mediated depletion of cellular α_v_ integrins and effect on FX mediated Ad5 trafficking

To evaluate the effect of depletion of cellular α_v_ integrins on FX mediated Ad5 trafficking, SKOV3 cells (5×10^4^ cells/well in either 24 well plates or 8 well chamber slides) were transfected with shRNA targeting αv integrin, “off-target” control shRNA, or liposomes only (mock transfected), according to manufacturer's instructions. Briefly, 2.5 µl/well of 5 µM shRNA was diluted 50 µl in serum free media before being mixed with 50 µl/well of SFDMEM containing 2 µl Dharmafect transfection reagent. The lipid and shRNA solution were mixed and allowed to stand at room temperature for 30 minutes before the addition of 400 µl/well of complete media. Cells were washed with PBS and 500 µl/well of lipid/shRNA solution was added and allowed to transfect cells for 24 hours prior to analysis of knockdown. We confirmed specific knockdown of α_v_ integrin by detection of α_v_ integrin mRNA by RT-qPCR and by flow cytometry for surface levels of the α_v_ subunit. Total cellular mRNA was harvested using RNeasy mini kit (Qiagen), quantified, and 300 ng of mRNA was converted to cDNA by *in vitro* reverse transcription. Levels of α_v_ integrin mRNA in 2.5 µl of cDNA were subsequently quantified by TAQman qPCR and normalised to total levels of the housekeeper GAPDH. For analysis of α_v_ integrin knockdown by flow cytometry, cells were detached 48 h post-transfection and incubated with an anti-α_v_ antibody (mAb mouse IgG_1_ clone L230) for 1 h at 4°C at a final concentration of 10 µg/ml. Cells were washed with SFDMEM, incubated with goat anti-mouse Alexa488-secondary (1∶125 dilution) for a further hour at 4°C, washed again with SFDMEM and resuspended in a final volume of 350µl. Surface levels of the α_v_ integrin subunit were detected using a BD FACS CANTO II flow cytometer, acquiring >10,000 gated events. For cell transport studies, cells were transfected as above in 8-well chamber slides for 24 hours. Cells were subsequently washed and cooled to 4°C, before the addition of fluorescently labelled Ad5 (10,000 vp/cell) in serum free media containing physiological levels of FX. Cells were then placed at 37°C for the stated times, washed, fixed using 4% paraformaldehyde in PBS for 10 minutes before counterstaining with 4′,6-diamidino-2-phenylindole (DAPI) and mounting in Prolong Gold for analysis as previously described.

### Analysis of Ad5 attachment *ex vivo*

Six µm frozen liver sections from macrophage-depleted male MF1 mice were incubated with 1×10^9^ vp of Alexa488-labelled Ad5CTL in SFDMEM in the presence or absence of 10 µg/ml FX and/or increasing concentrations of heparins for 1 h on ice. Sections were then washed twice with PBS and mounted using ProLong Gold antifade reagent with DAPI. Sections were imaged using an Olympus Cell∧M imaging system. To quantify adherent Alexa488-Ad5CTL particles, 40× images captured using an Olympus imaging system and were processed using PaintShop Pro and ImageJ. Viral particles were counted using the semi-automated cell counting tool from ImageJ. An average of 5 captured images were analysed per experimental condition.

### *In vivo* assessment of Ad5 uptake

All animal experiments were approved by the UK Home Office. Male MF1 outbred mice aged between 8–10 weeks (weight approximately 35g) and housed in secure barrier facilities were used for all *in vivo* experiments. Macrophage depletion was carried out by clodronate liposome pretreatment as described previously [21], [66]. Kupffer cell depletion was confirmed by staining frozen liver sections with a rat anti-mouse F4/80 primary antibody at a 1∶50 dilution (Abcam, Cambridge, UK) and a goat anti-rat Alexa Fluor 546 (red) secondary antibody at a 1∶500 dilution and all sections in macrophage depleted mice showed a complete absence of Kupffer cells to confirm the efficiency of depletion (data not shown). For the analysis of virus genome accumulation in the liver 1 h after intravenous virus administration, 1×10^11^ vp Ad5CTL in 100 µl PBS was injected into the tail-vein of macrophage-depleted mice 5 minutes after intravenous administration of 20 mg/kg or 50 mg/kg heparins in 100 µl PBS. Mice were sacrificed and perfused with PBS 1h post-inoculation. Livers were harvested and total DNA was purified using the QiaQuick Spin DNA Extraction Kit as described above.

### Analysis of Ad5 localisation *in vivo*

To characterise Ad localisation *in vivo*, 1×10^11^ vp Alexa-labelled Ad5CTL in 100 µl PBS was injected into the tail vein 5 minutes after intravenous administration of 20 mg/kg or 5 0mg/kg heparins in 100 µl PBS. Livers were flushed by cardiac PBS perfusion 1 h later to remove non-adherent virus particles and the largest lobe was then embedded and immediately frozen in OCT Tissue-Tek embedding compound. Frozen liver sections (4 µm) were fixed and stained with rat anti-mouse CD31 antibody at a 1∶50 dilution (BD Pharmingen, Oxford, UK) to detect endothelial cells. Specific binding of primary antibodies was visualised using a goat anti-rat Alexa Fluor 546 (red) secondary antibody in PBS at a dilution of 1∶500. Sections were imaged using an Olympus imaging system.

### Statistical analysis

Statistical significance was calculated using 2-sample, 2-tailed student's *t*-tests. P-values of <0.05 or over were considered statistically significant. Results presented are representative data from a minimum of two separate experiments with at least 3 experimental replicates per group. All virus binding and transduction experiments were performed in biological triplicates and on at least three independent occasions. All error bars represent SEM.

## Supporting Information

Figure S1 Analysis of heparinase III pretreatment *in vitro*. (**A**) The expression of intact heparan sulfate (using the 10E4 antibody) or heparinase III-digested heparan sulfate ‘stubs’ (using the 3G10 antibody) was analysed by flow cytometry in SKOV3 cells that had been treated with increasing doses of heparinase III (0 U/ml, 0.5 U/ml, 1 U/ml or 5 U/ml) for 1 h at 37°C. Each marker was tested on at least 3 independent samples and raw traces as well as quantitative data are shown. Data are presented as average % of expressing/positive cells +/− SEM. (**B**) The expression of chondroitin sulfate was analysed by flow cytometry in SKOV3 cells that had been treated with increasing doses of heparinase III or chondroitinase ABC (0 U/ml, 0.5 U/ml, 1 U/ml or 5 U/ml). At least 3 independent samples were tested and data are presented as average % of expressing/positive cells +/− SEM. (**C+D**) SKOV3 cells that had been pretreated with increasing doses of heparinase III (HepIII) for 1 h at 37°C were transduced with 1000 vp/cell of Ad5 in the presence or absence of 10 µg/ml FX. (**C**) Binding of 1000 vp/cell Ad5CTL to SKOV3 cells was quantified after incubation with cells for 1 h at 4°C as described previously. (**D**) Adenoviral gene transfer was quantified after incubation of 1000 vp/cell Ad5CTL with SKOV3 cells for 1 h or 3 h at 37°C. β-galactosidase expression was quantified 48 h post-transduction and normalised to total protein content. *p<0.05, **p<0.01 compared to control. Error bars represent S.E.M.
(1.19 MB TIF)

Figure S2 FX-mediated enhancement of gene transfer in CAR^low^ and CAR^high^ cell lines. SKOV3, MDA-MB-231, A549, SNB19, HT29 or NCI-H522 cells were transduced with 1000 vp/cell of Ad5CTL (closed bars) or Ad5PD1 (open bars) in the presence and absence of 10 µg/ml FX for 3 h at 37°C. Results are shown as FX-mediated fold-enhancement over control conditions (virus alone). Reporter gene expression was quantified 48 h post-transduction as described previously. Analysis of each cell type was performed at least on 3 independent occasions and at least in triplicate within each experiment.
(0.08 MB TIF)

Figure S3 Transport of fluorescently-labelled Ad5CTL and Ad5KO1 in SKOV3 cells. 10,000 vp/cell of Alexa-labelled Ad5CTL or Ad5KO1 were allowed to bind cells for 1 h at 4°C in the presence or absence of 10 µg/ml FX. Cells were then incubated at 37°C for 0 min to 60 min prior to fixation. Nuclei were counterstained using DAPI. Images were captured using a 60× microscope objective.
(2.72 MB TIF)

Figure S4 Transport of fluorescent-labelled Ad5 in the presence of PKA, PI3K and p38MAPK inhibitors. (A) A549 cells were incubated with PKA, P13K or p38MAPK inhibitors for 30 min at 37°C then 10,000 vp/cell of Alexa488-labelled Ad5CTL (green particles) in the presence of FX and the different kinase inhibitors were allowed to bind cells for 1 h at 4°C, followed by incubation at 37°C for 3 h to allow internalisation and intracellular transport prior to fixation and staining for the MTOC marker pericentrin (red). Nuclei were counterstained using DAPI. The PKA inhibitor H 89 dihydrochloride, the PI3K inhibitor LY 294002 hydrochloride and the p38MAPK inhibitor SB 203580 hydrochloride were used. Representative images are shown. (B) Percentage of cells with colocalisation of fluorescently-labelled Ad5CTL with the MTOC marker pericentrin in A549 cells was calculated by analysing at least 5 separate 40× microscope fields per experimental condition. ** = p<0.01 compared to Ad5CTL+FX values.
(0.68 MB TIF)

Figure S5 Effect of sodium chlorate on Ad5CTL-mediated cell binding and gene transfer. (**A**) Sulfated glycosaminoglycan (sGAG) content of cultured SKOV3 cells pretreated with increasing concentrations of sodium chlorate (0 mM, 20 mM, 50 mM) was measured using the Blyscan sulfated GAG assay kit, which quantitates specific binding of the sulfate-binding cationic dye, 1, 9-dimethylmethylene blue. (**B**) Binding of 1000 vp/cell Ad5CTL to SKOV3 cells pretreated with increasing concentrations of sodium chlorate (0 mM, 20 mM, 50 mM) was quantified after incubation with cells for 1 h at 4°C as described previously. (**C**) MTT assay assessment of cell viability in the presence of increasing concentrations of sodium chlorate.
(0.14 MB TIF)

Table S1 IC_50_ values of heparins/heparan sulfate for CAR- or α_v_ integrin binding-mutant adenoviruses (µg/ml). SKOV3 cells were transduced with 1000 vp/cell of Ad5KO1 or Ad5PD1 in the presence or absence of 10 µg/ml FX and varying concentrations of heparins/heparan sulfates for 3 h at 37°C. Reporter gene expression was quantified 48 h post-transduction as described previously. IC_50_ values were calculated using the Hill-Slope model.
(0.03 MB DOC)
