# Supplementary figures and images for: Requirements for Receptor Engagement during Infection by Adenovirus Complexed with Blood Coagulation Factor X
Source: PLoS Pathog. 2010 Oct 7;6(10):e1001142. doi: 10.1371/journal.ppat.1001142 (PMC2951380; doi:10.1371/journal.ppat.1001142)

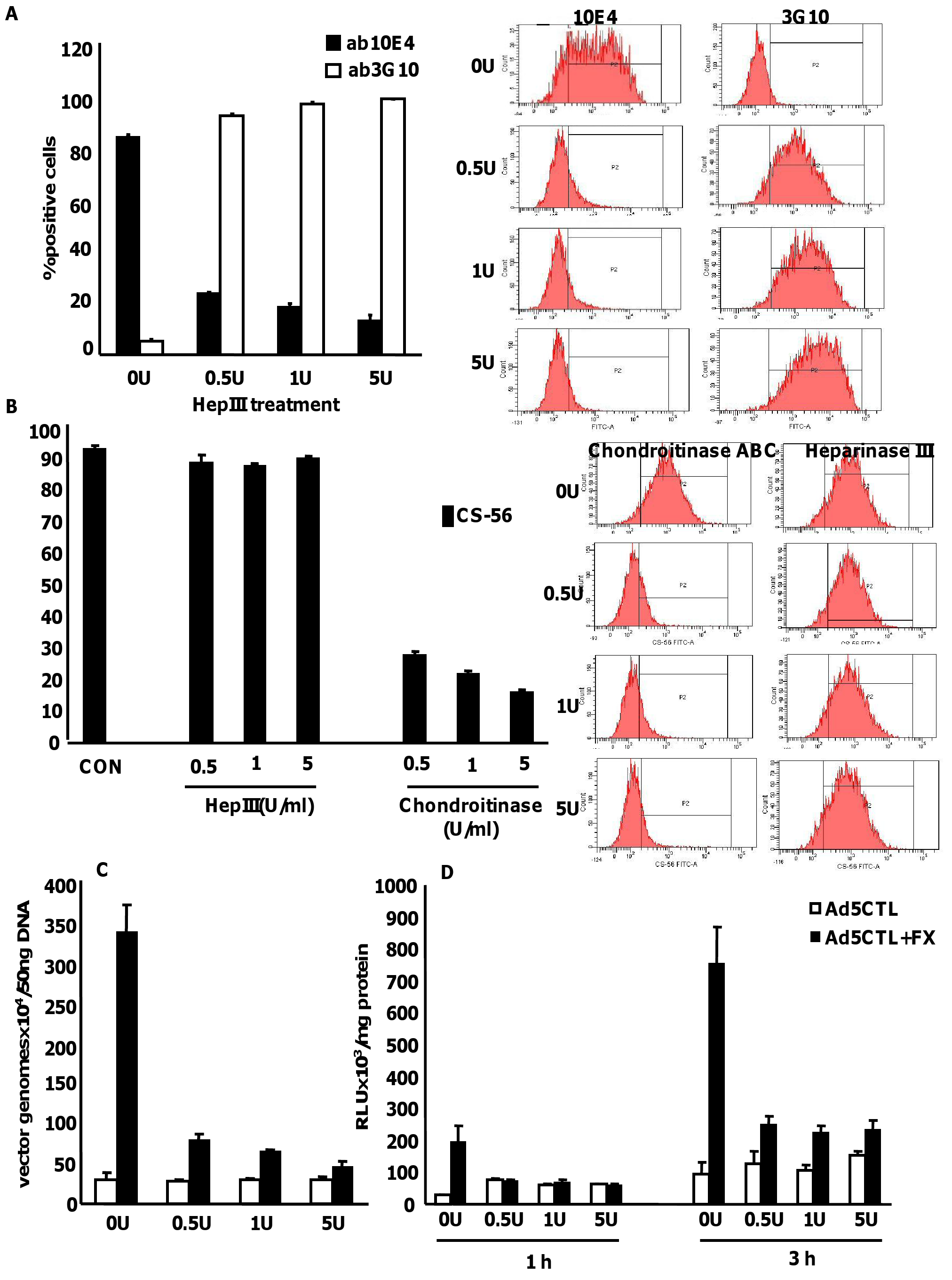

Supplement: Figure S1 — Analysis of heparinase III pretreatment in vitro. (A) The expression of intact heparan sulfate (using the 10E4 antibody) or heparinase III-digested heparan sulfate ‘stubs’ (using the 3G10 antibody) was analysed by flow cytometry in SKOV3 cells that had been treated with increasing doses of heparinase III (0 U/ml, 0.5 U/ml, 1 U/ml or 5 U/ml) for 1 h at 37°C. Each marker was tested on at least 3 independent samples and raw traces as well as quantitative data are shown. Data are presented as average % of expressing/positive cells +/− SEM. (B) The expression of chondroitin sulfate was analysed by flow cytometry in SKOV3 cells that had been treated with increasing doses of heparinase III or chondroitinase ABC (0 U/ml, 0.5 U/ml, 1 U/ml or 5 U/ml). At least 3 independent samples were tested and data are presented as average % of expressing/positive cells +/− SEM. (C+D) SKOV3 cells that had been pretreated with increasing doses of heparinase III (HepIII) for 1 h at 37°C were transduced with 1000 vp/cell of Ad5 in the presence or absence of 10 µg/ml FX. (C) Binding of 1000 vp/cell Ad5CTL to SKOV3 cells was quantified after incubation with cells for 1 h at 4°C as described previously. (D) Adenoviral gene transfer was quantified after incubation of 1000 vp/cell Ad5CTL with SKOV3 cells for 1 h or 3 h at 37°C. β-galactosidase expression was quantified 48 h post-transduction and normalised to total protein content. *p<0.05, **p<0.01 compared to control. Error bars represent S.E.M. (1.19 MB TIF) [file ppat.1001142.s001.tif]

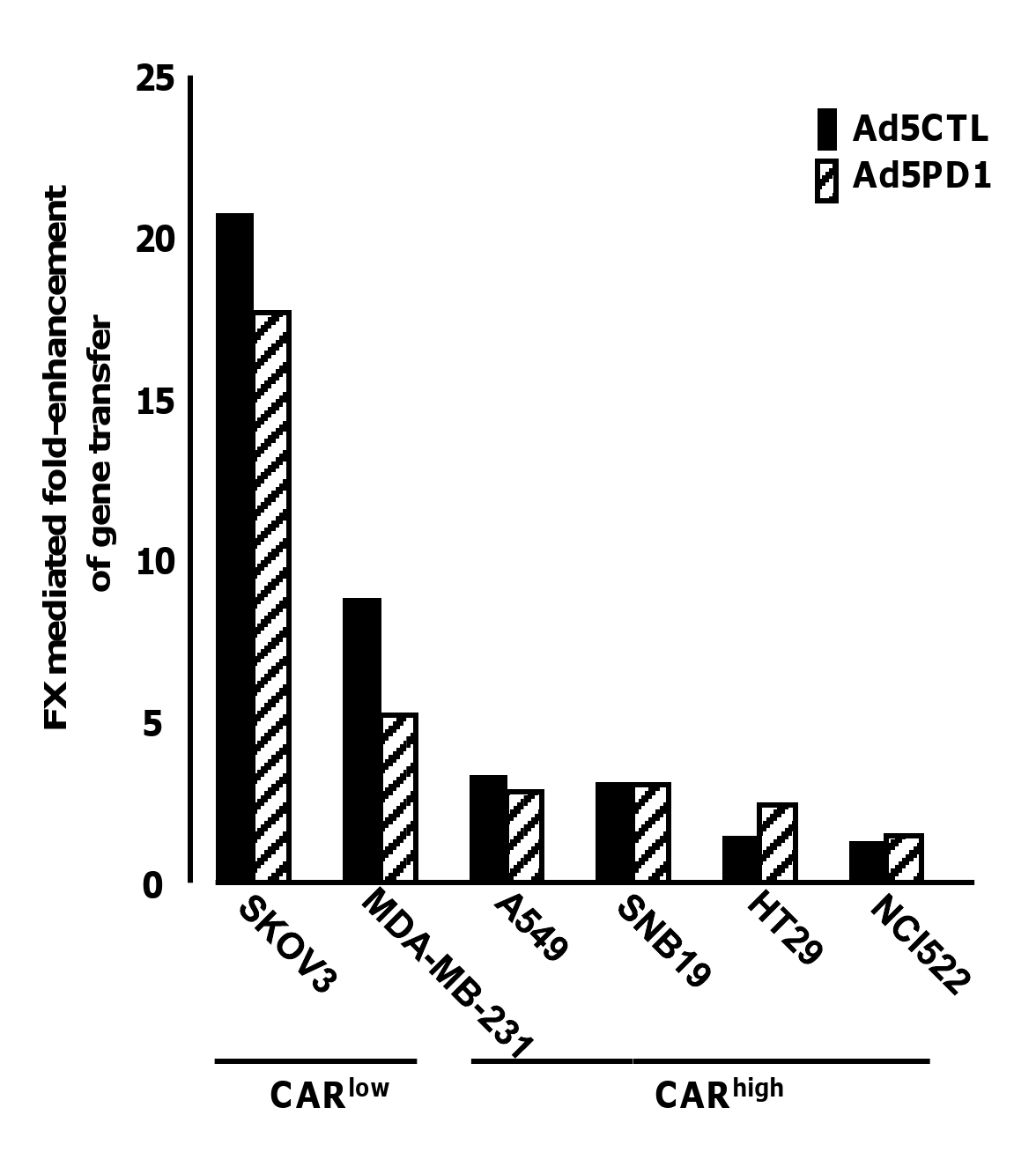

Supplement: Figure S2 — FX-mediated enhancement of gene transfer in CARlow and CARhigh cell lines. SKOV3, MDA-MB-231, A549, SNB19, HT29 or NCI-H522 cells were transduced with 1000 vp/cell of Ad5CTL (closed bars) or Ad5PD1 (open bars) in the presence and absence of 10 µg/ml FX for 3 h at 37°C. Results are shown as FX-mediated fold-enhancement over control conditions (virus alone). Reporter gene expression was quantified 48 h post-transduction as described previously. Analysis of each cell type was performed at least on 3 independent occasions and at least in triplicate within each experiment. (0.08 MB TIF) [file ppat.1001142.s002.tif]

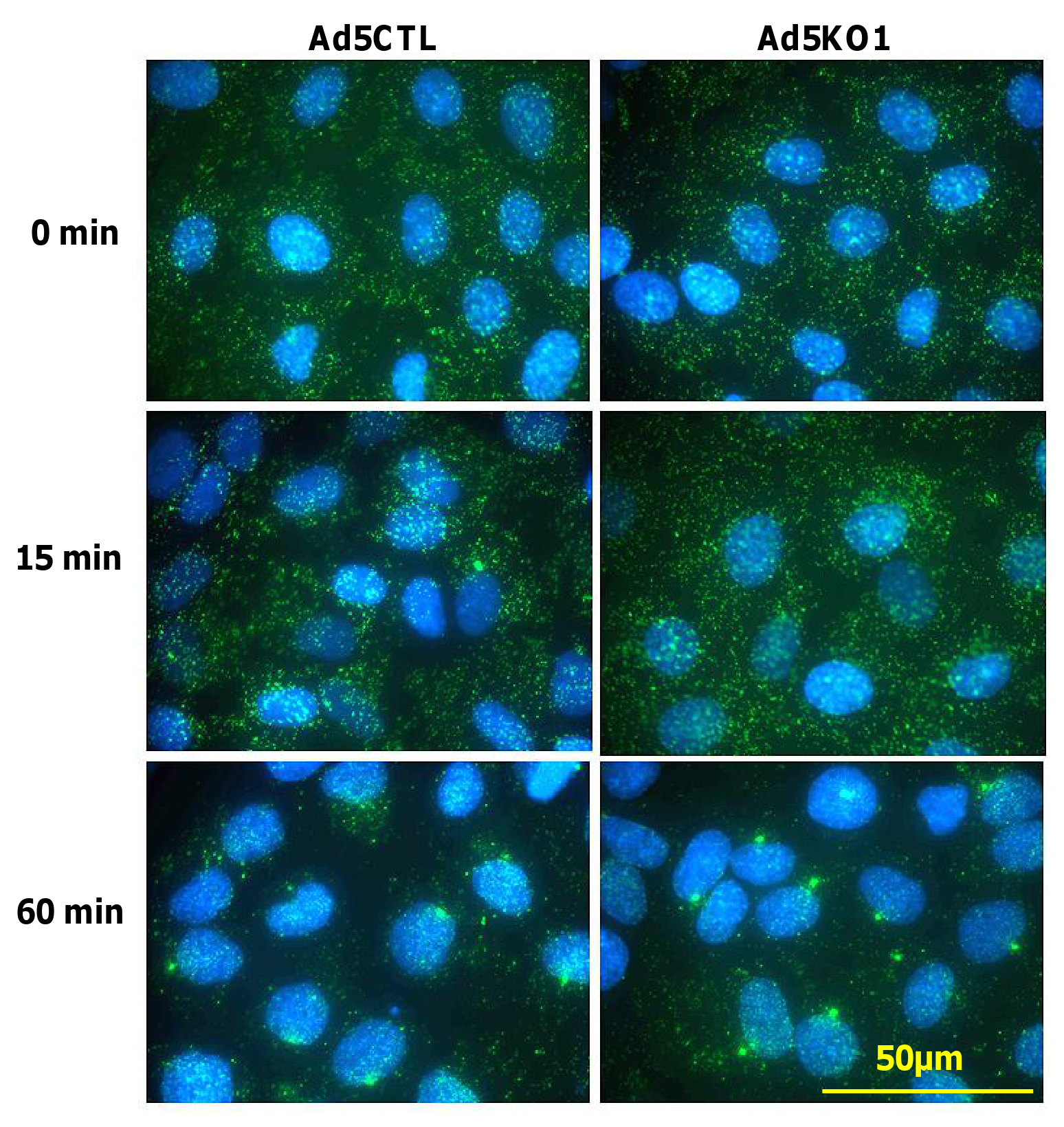

Supplement: Figure S3 — Transport of fluorescently-labelled Ad5CTL and Ad5KO1 in SKOV3 cells. 10,000 vp/cell of Alexa-labelled Ad5CTL or Ad5KO1 were allowed to bind cells for 1 h at 4°C in the presence or absence of 10 µg/ml FX. Cells were then incubated at 37°C for 0 min to 60 min prior to fixation. Nuclei were counterstained using DAPI. Images were captured using a 60× microscope objective. (2.72 MB TIF) [file ppat.1001142.s003.tif]

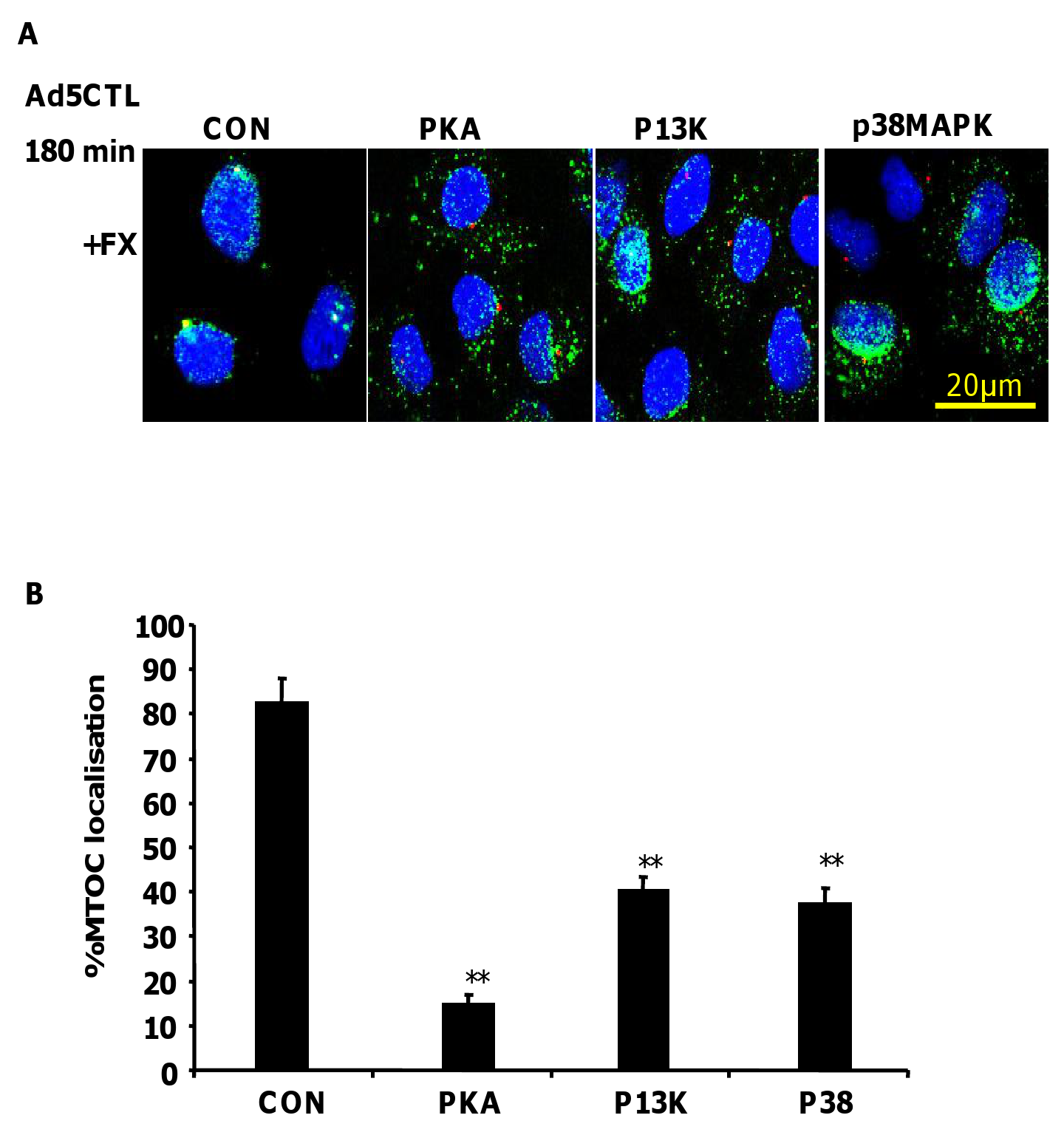

Supplement: Figure S4 — Transport of fluorescent-labelled Ad5 in the presence of PKA, PI3K and p38MAPK inhibitors. (A) A549 cells were incubated with PKA, P13K or p38MAPK inhibitors for 30 min at 37°C then 10,000 vp/cell of Alexa488-labelled Ad5CTL (green particles) in the presence of FX and the different kinase inhibitors were allowed to bind cells for 1 h at 4°C, followed by incubation at 37°C for 3 h to allow internalisation and intracellular transport prior to fixation and staining for the MTOC marker pericentrin (red). Nuclei were counterstained using DAPI. The PKA inhibitor H 89 dihydrochloride, the PI3K inhibitor LY 294002 hydrochloride and the p38MAPK inhibitor SB 203580 hydrochloride were used. Representative images are shown. (B) Percentage of cells with colocalisation of fluorescently-labelled Ad5CTL with the MTOC marker pericentrin in A549 cells was calculated by analysing at least 5 separate 40× microscope fields per experimental condition. ** = p<0.01 compared to Ad5CTL+FX values. (0.68 MB TIF) [file ppat.1001142.s004.tif]

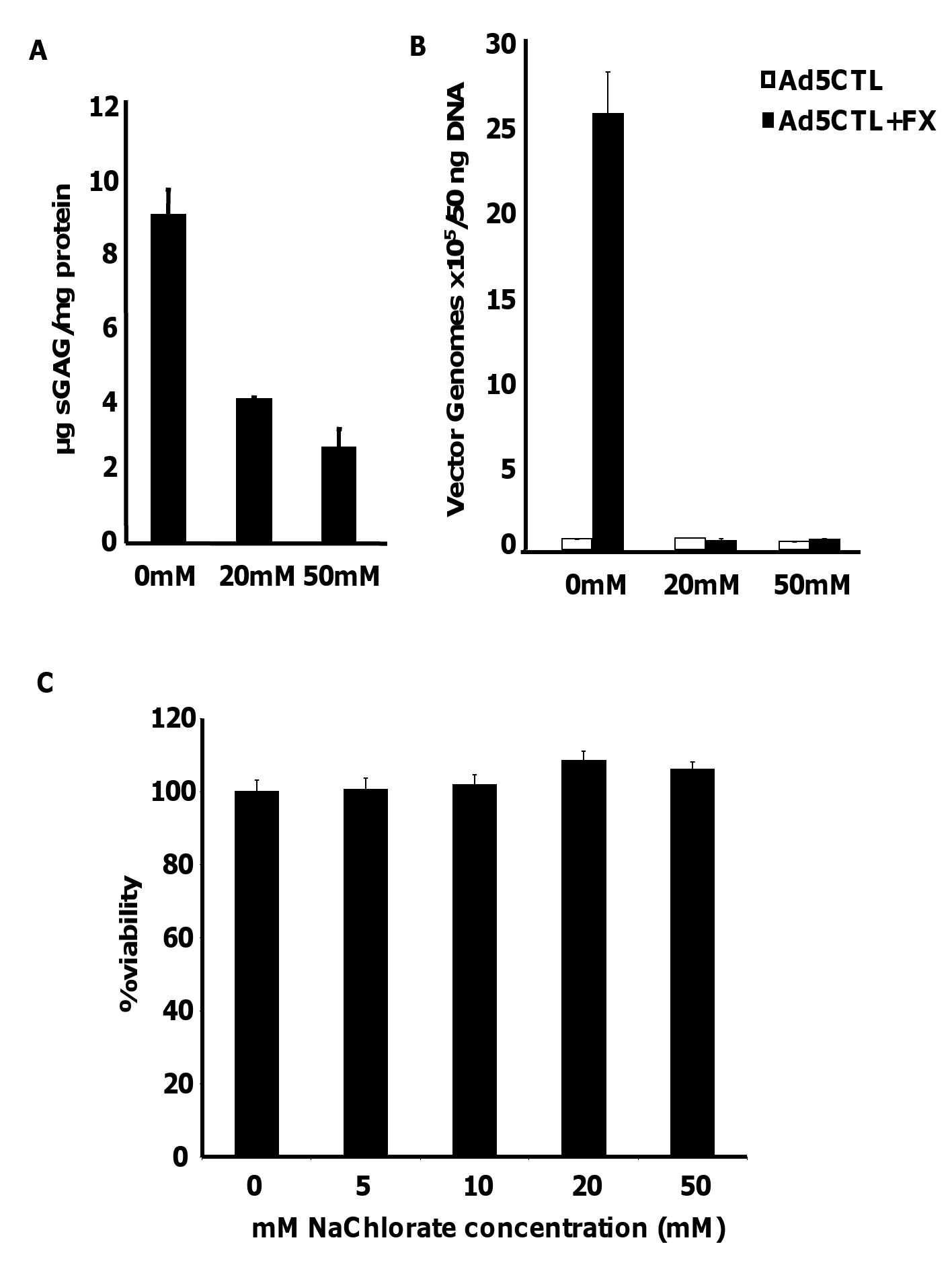

Supplement: Figure S5 — Effect of sodium chlorate on Ad5CTL-mediated cell binding and gene transfer. (A) Sulfated glycosaminoglycan (sGAG) content of cultured SKOV3 cells pretreated with increasing concentrations of sodium chlorate (0 mM, 20 mM, 50 mM) was measured using the Blyscan sulfated GAG assay kit, which quantitates specific binding of the sulfate-binding cationic dye, 1, 9-dimethylmethylene blue. (B) Binding of 1000 vp/cell Ad5CTL to SKOV3 cells pretreated with increasing concentrations of sodium chlorate (0 mM, 20 mM, 50 mM) was quantified after incubation with cells for 1 h at 4°C as described previously. (C) MTT assay assessment of cell viability in the presence of increasing concentrations of sodium chlorate. (0.14 MB TIF) [file ppat.1001142.s005.tif]
