# Supplementary material for: Requirements for Receptor Engagement during Infection by Adenovirus Complexed with Blood Coagulation Factor X
Source: PLoS Pathog. 2010 Oct 7;6(10):e1001142. doi: 10.1371/journal.ppat.1001142 (PMC2951380; doi:10.1371/journal.ppat.1001142)
Supplement: Table S1 — IC50 values of heparins/heparan sulfate for CAR- or αv integrin binding-mutant adenoviruses (µg/ml). SKOV3 cells were transduced with 1000 vp/cell of Ad5KO1 or Ad5PD1 in the presence or absence of 10 µg/ml FX and varying concentrations of heparins/heparan sulfates for 3 h at 37°C. Reporter gene expression was quantified 48 h post-transduction as described previously. IC50 values were calculated using the Hill-Slope model. (0.03 MB DOC) [file ppat.1001142.s006.doc]

| **Heparin analogue** | **Ad5KO1**  **IC50** | **Ad5PD1**  **IC50** |
| --- | --- | --- |
| De-*O*-sulfated heparin | 111.72 | 97.61 |
| De-*N*-sulfated heparin | 51.29 | 52.55 |
| Bovine intestinal heparin | 4.06 | 8.83 |
| Porcine intestinal heparan sulfate | 4.22 | 7.72 |
